# The full dynamics of energy relaxation in large organic molecules: from photo-excitation to solvent heating†
†Electronic supplementary information (ESI) available. See DOI: 10.1039/c9sc00410f


**DOI:** 10.1039/c9sc00410f

**Published:** 2019-04-02

**Authors:** Vytautas Balevičius Jr, Tiejun Wei, Devis Di Tommaso, Darius Abramavicius, Jürgen Hauer, Tomas Polívka, Christopher D. P. Duffy

**Affiliations:** a School of Chemical and Biological Sciences , Queen Mary University of London , Mile End Road , London E1 4NS , UK . Email: c.duffy@qmul.ac.uk; b Institute of Chemical Physics , Vilnius University , Sauletekio av. 9 , Vilnius , LT-10222 , Lithuania; c Fakultät für Chemie , Technical University of Munich , Lichtenbergstraße 4 , D-85748 Garching , Germany; d Photonics Institute , TU Wien , Gußhausstraße 27 , 1040 Vienna , Austria; e Institute of Physics and Biophysics , Faculty of Science , University of South Bohemia , Branišovská 1760 , 37005 České Budějovice , Czech Republic

## Abstract

In some molecular systems, such as nucleobases, polyenes or the active ingredients of sunscreens, substantial amounts of photo-excitation energy are dissipated on a sub-picosecond time scale, raising questions such as: where does this energy go or among which degrees of freedom it is being distributed at such early times? Here we use transient absorption spectroscopy to track excitation energy dispersing from the optically accessible vibronic subsystem into the remaining vibrational subsystem of the solute and solvent. Monitoring the flow of energy during vibrational redistribution enables quantification of local molecular heating. Subsequent heat dissipation away from the solute molecule is characterized by classical thermodynamics and molecular dynamics simulations. Hence, we present a holistic approach that tracks the internal temperature and vibronic distribution from the act of photo-excitation to the restoration of the global equilibrium. Within this framework internal vibrational redistribution and vibrational cooling are emergent phenomena. We demonstrate the validity of the framework by examining a highly controversial example, carotenoids. We show that correctly accounting for the local temperature unambiguously explains their energetically and temporally congested spectral dynamics without the *ad hoc* postulation of additional ‘dark’ states. An immediate further application of this approach would be to monitor the excitation and thermal dynamics of pigment–protein systems.

## Abstract 2

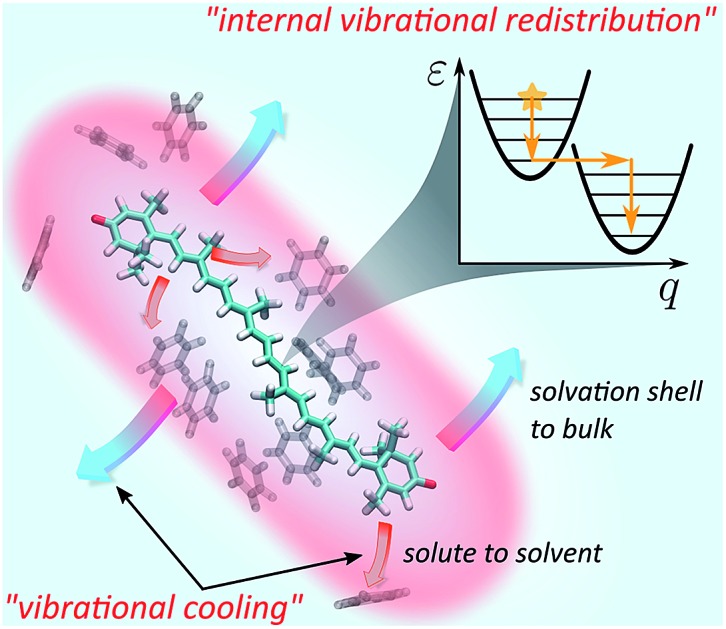
In some molecular systems, such as nucleobases, polyenes or sunscreens, substantial amounts of photo-excitation energy are dissipated on a sub-picosecond time scale. Where does this energy go or among which degrees of freedom it is being distributed at such early times?

## 1 Introduction

Photoexcitation of molecules in solution immediately launches a cascade of relaxation processes: solvent relaxation, internal conversion (IC), vibrational energy relaxation and any possible photo-chemical reactions, such as photo-isomerisation, to name but a few.^1^,^2^ Even restricting to the simplest and seemingly trivial scenario of sequential electronic relaxation, determining and quantifying the energy relaxation pathways for large molecules is a daunting task due to the sheer number of participating degrees of freedom. One common simplification based on the separation of time scales is the division of vibrational relaxation into two distinct processes: internal vibrational redistribution (IVR) and vibrational cooling (VC). The former process represents the relaxation of a small sub-set of optically active vibrations following their initial excitation. Meanwhile, VC represents the flow of energy from the solute to the solvent molecules. It is typically assumed that the two processes are sequential, which underlies the separation itself. Namely, IVR leads to the redistribution of electronic excitation energy over the whole vibrational sub-system and effectively establishes a quasi-equilibrium corresponding to an elevated local temperature. Subsequently, the cooling of the thermal vibrations, VC, takes place as heat dissipation into the immediate environment. However, this separation is sometimes questionable and the interplay of these processes is important in numerous areas of chemical physics, ranging from heat transfer in proteins and nano-structures^3^ and thermal conduction though molecular junctions^4^ to chemical reaction mechanisms.^5^

Understanding the vibrational relaxation rates and pathways becomes important when they fall into the temporal domain of other processes,^1^ such as bond breaking and formation during chemical reactions^6^ or energy transfer in pigment–protein complexes.^7^ There have been multiple studies aimed at both IVR and VC in either the first excited state, S_1_, or the ground state, S_0_. Earlier studies explored the principles of cooling on the relatively long-lived S_1_ state in the laser dyes based on coumarins,^8^,^9^ stilbenes,^10^–^12^ malachite green,^13^ oxazine and rhodamine.^14^ On the other hand, in some systems the lifetime of S_1_ state is remarkably short, the prime examples of such systems being nucleobases,^15^ solar screen molecules,^16^ polyenes^17^ and carotenoids.^18^ At the same time, the absorption spectra of these systems are lying in the visible or UV, which means that tremendous amounts of excitation energy are being disposed of on breathtakingly short time scales (as much as 2.1 eV in carotenoids^19^ or 4 eV in nucleobases^20^ in times as short as 200 fs). This raises questions such as: where exactly does the disposed energy go? Or what are the effects that such ultra-fast disposal has on the molecules and their associated observables? Fundamental understanding of these questions is important because of the biological relevance of the mentioned molecules: from the role of nucleoside resilience to photodamage in early life evolution^21^ to carotenoid photo-protection of light-harvesting proteins^22^ to biomedical application of photo-protection by sunscreens.^23^

**Fig. 1:**
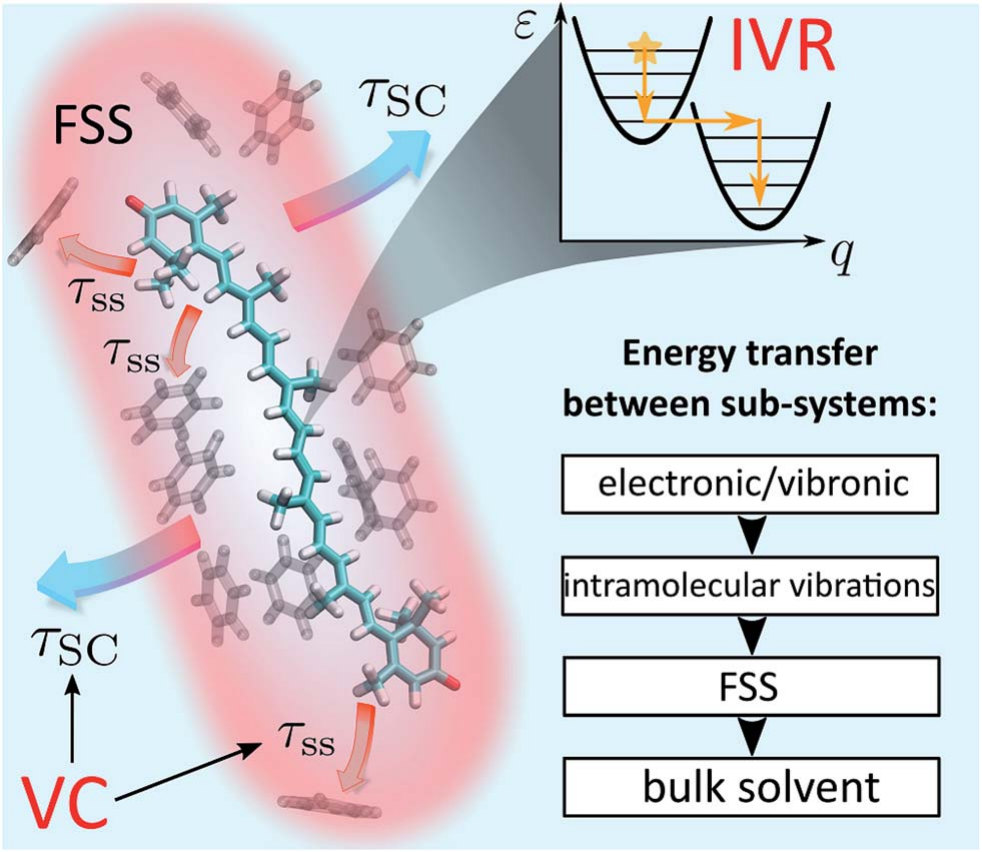
Schematic representation of IVR and VC processes in solution. The event of photoexcitation instantaneously promotes the molecule to a non-equilibrium state and launches the IVR cascade within the high-frequency optically active modes. Subsequently, energy exchange between the molecule and the solvent takes place. In a coarse-grained picture, this exchange can be interpreted as energy transfer from the solute to the first solvation shell (FSS; shown in red) and heat dissipation from the FSS to the bulk solvent. The latter processes constitute VC and are characterized by time scales *τ*_ss_ (“solute-to-solvent”) and *τ*_SC_ (“shell cooling”), respectively. The inset summarizes the conceptual scheme of energy relaxation *via* successive layers.

Even though IVR and VC of molecules in solution have been subjects of extensive research,^24^–^27^ the results to date are not general and conclusive. The relative simplicity of small molecules (2–3 atoms) allows for truly detailed mapping of internal energy relaxation.^27^,^28^ Intermediate size molecules with a number of atoms between 10–30, such as various dyes, are comparatively well studied as well. In particular, several prominent studies of azulene^29^,^30^ and *trans*-stilbene^12^ report various mechanisms of IVR and VC, presenting a coarse-grained description in which the solvent is partitioned into the first solvation shell^31^ (FSS) and the bulk, Fig. 1. Within such a picture, VC is a composite process consisting of heat transfer from the solute to FSS, and subsequently from FSS to the bulk solvent. Lastly, there have been several analyses of relaxation mechanisms in truly large molecules, such as porphyrins^32^ or hemes in proteins,^13^,^33^ however, a universal theory for IVR and VC in intermediate and large molecules in liquids is still lacking.^1^ An appealing alternative is the non-equilibrium molecular dynamics (MD) approach.^34^–^36^ It offers a route that avoids complicated and possibly excessive parametrization of specific rates. However, an additional task remains to connect such studies to particular experimental observables rather than just the characteristic time scales.

The goal of this study is to formulate a general dynamical framework of optical excitation energy conversion into thermal energy residing both within the molecule and flowing out into the solvent. While the distinction between IVR and VC is traditionally based on the separation of their respective time scales, we do not resort to this *ad hoc* classification. Instead we quantum-mechanically model IVR as internal solute dynamics starting from a vibronic Hamiltonian. Conversely, VC is modeled classically by introducing the concept of local temperature. The solvent is partitioned into FSS and the bulk, Fig. 1, as suggested earlier, whereas an MD simulation is employed to determine the FSS and consequently estimate the time scale of heat diffusion from FSS to the bulk. Our unified treatment ensures that the system and local temperature evolutions are modeled self-consistently. Ultimately, we model the optical response of the solute in terms of transient absorption (TA) spectra, which elucidates the internal heating effects and identifies signatures of the IVR and VC contributions. We learn that IVR-related effects are present on much later timescales than one would expect, up to tens of picoseconds. As a particularly relevant example, the photodynamics of carotenoids are analyzed. Being members of the polyene family, carotenoids posses a complicated structure of short-lived electronic states.^18^ This has led to multiple conjectures regarding the observed spectroscopic signals.^37^ Particularly, an absorptive feature termed S* is sometimes referred to as an optically dark electronic state.^37^ Instead we shown how this feature comes about as a result of peculiar vibronic and temperature dynamics, and reproduce the evolution of carotenoid TA spectra from hundreds of femtoseconds to tens of picoseconds. This demonstrates that the presented treatment enables modeling of large polyatomic molecules in a way that disentangles the concomitant relaxations processes.

This manuscript is organized as follows: in Section 2 we present the theoretical foundations of the manuscript (following the summary shown in the inset of Fig. 1). We start with the description of relaxation within a vibronic subsystem (Subsection 2.1). We then discuss the energy redistribution within the intramolecular normal modes and subsequent transfer to the FSS and still further to the bulk solvent (Subsection 2.2). To quantify these processes we introduce the concept of effective local temperature. Next, we provide a prescription for monitoring the system dynamics by TA spectroscopy (Subsection 2.3), and introduce particular model and procedures for the implementation of the presented methodology (Subsection 2.4). In Section 3 we present the results, first focusing on an abstract model system to demonstrate the key features of the methodology (Subsection 3.1), then modeling the full dynamics of two carotenoid solutions as a particular application of interest (Subsection 3.2). Finally, in Section 4 Conclusions and outlook are presented.

## 2 Theory and model

### 2.1 Excitation relaxation in a vibronic system

In many large molecules several vibrational modes are optically active. Therefore we will consider the evolution of excitation in a vibronic subsystem, where the vibronic states are quantum states that require both the electronic and vibrational indices for their characterization. There are several approaches to model vibrational energy relaxation with explicit treatment of the vibronic states, ranging from rigorously quantum mechanical^38^,^39^ to semi-phenomenological.^40^,^41^ Here, we employ an intermediate scheme suitable for modeling optical response of strongly-pronounced vibronic transitions, termed the vibrational energy relaxation approach.^42^ It treats population dynamics *via* quantum mechanical master equations and models the TA spectra in a semi-phenomenological fashion, and it has been shown to reproduce numerous TA features of carotenoids in particular with high accuracy.^43^,^44^

Let us outline this scheme by considering a manifold of electronic states of a molecule linearly coupled to its vibrational subsystem. The following approach rests upon the observation that typically only a limited set of high-frequency vibrational modes strongly couple to the optical transitions in molecules. These high-frequency modes require explicit treatment and corresponding optical transitions should be considered in terms of vibronic states. The remaining modes can be treated either classically or implicitly as a thermal bath. A good criterion for such a separation is the temperature: the set of high-frequency under-damped modes (*ħω* ≫ *kT*) can then be quantized as harmonic oscillators, while all the low-frequency (*ħω* < *kT*) modes constitute the thermal bath. To describe the relaxation processes within the given molecular system, in this paper we consider the following Hamiltonian of electronic states |*i*〉 with energies with energies *ε*_*i*_ coupled to a finite set of harmonic oscillators, *α* = 1, …*K*, *via* mutually shifted potential energy surfaces:
1

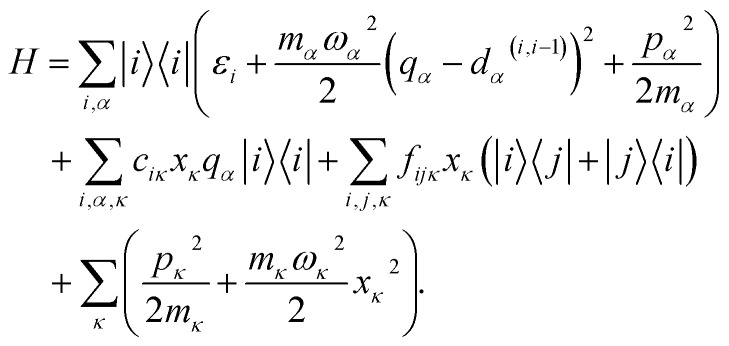

Here, *q*_*α*_ and *p*_*α*_ denote the coordinate and momentum of the *α*-th vibrational mode (oscillator of mass *m*_*α*_ and frequency *ω*_*α*_), and *d*_*α*_^(*ij*)^ denotes the mutual displacement of this mode's potentials between states |*i*〉 and | and |*j*〉. The modes are coupled with strength . The modes are coupled with strength *c*_*iκ*_ to an infinite set of harmonic oscillators of the bath with coordinates *x*_*κ*_ and momenta *p*_*κ*_. Several approximations are made here. Firstly, we assume that the same normal modes are associated with each electronic state, which is not strictly true due to vibronic interactions and differences in potential energy surfaces (conf., Duschinsky rotations^45^). To make the model simpler, the coupling strengths *c*_*iκ*_ are set identical for all modes associated with the same electronic state. Secondly, the off-diagonal elements of the electronic subspace are coupled to the oscillators of the bath with coupling strengths *f*_*ijκ*_, which means that such coupling is vanishing at equilibrium 
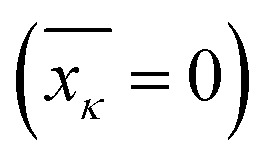
 and affects the system dynamics only *via* fluctuations. Such a treatment disregards the fact that some electronic states can be coupled non-adiabatically (*i.e.*, conical intersections), these effects could be introduced separately.

By quantizing the high-frequency system modes, we recast the Hamiltonian into the vibronic basis spanned by the states |*i*_*a*_〉 = | = |*i*〉||*a*_1_〉^*i*^…|*a*_*K*_〉^*i*^, where |*a*_*α*_〉^*i*^ are the wave-functions of the high-frequency modes on the *i*-th electronic state. We employ a tuple *a* = (*a*_1_, …, *a*_*α*_, …, *a*_*K*_) as a bookkeeping devise, where the entries indicate the number of quanta of the *α*-th mode with energy spacing *ω*_*α*_ within the associated electronic state. We also refer to all the vibronic states (combinations of *a*) associated with the electronic state |*i*〉 as a vibronic manifold. The Hamiltonian as a vibronic manifold. The Hamiltonian eqn (1) recast into the vibronic basis is given in the ESI.† The dynamics of the vibronic states |*i*_*a*_〉 are described by the equations of motion for the corresponding populations are described by the equations of motion for the corresponding populations *n*_*a*_^*i*^(*t*). The equations are derived from the quantum Liouville equation under the secular and Markov approximations^42^,^46^ and are given in the ESI.† Essentially, they are of the second order with respect to the system–bath couplings, *c*_*iκ*_/*f*_*ijκ*_, which are characterized by the spectral densities 
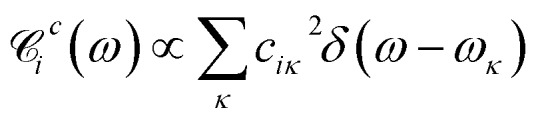
 and 
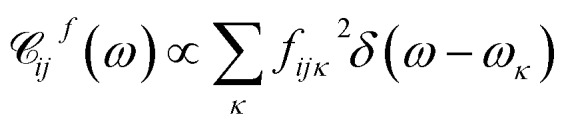
. A particular form of the spectral density should be chosen to reflect the specific physical situation under consideration. In principle, the spectral densities include both the intramolecular degrees of freedom (low-frequency modes) and the solvent phonons that couple to the electronic or vibronic degrees of freedom. This effectively yields a master equation, with Fermi golden rule-type transition rates between the vibronic states. Namely, the rates are proportional to the spectral densities 𝒞(*ω*) probed at the energy gaps of the transitions. Additionally, the rates within a vibronic manifold are proportional to the respective vibrational quantum numbers, while the inter-manifold transitions are weighted by corresponding Franck–Condon factors, *F*_*ia*,*ja*′_^*α*^ = ^*i*^〈*a*_*α*_|*a*_*α*_′′〉^*j*^. We note that by virtue of detailed balance in the bath correlation functions, the presented model has readily inbuilt thermodynamics.

### 2.2 Local molecular heating and cooling

The majority of formal excitation energy relaxation studies consider a molecule or an assembly of molecules to be in contact with an infinite and infinitely fast heat reservoir. Indeed such a depiction is reasonable insofar as the IC and the associated vibrational relaxation are the limiting steps when compared to the inter-molecular energy redistribution. However, in some situations this assumption can be questionable. *E.g.*, when ultrafast ICs are involved, the energy dissipation to the medium might not appear as instantaneous and the finite vibrational energy transfer between molecules should be considered. Additionally, a quasi-equilibrium of intra-molecular vibrations can be established in a situation where molecule-to-medium relaxation is the rate limiting step. This quasi-equilibrium gives rise to the effective local temperature.^47^ Let us consider the vibrational subsystem of a molecule. This subsystem can be considered the immediate recipient of the energy given away by the electronic subsystem during IC: after all, a large molecule is its own immediate bath. In such a case, to quantify the redistribution of energy we turn to the internal energy of the molecule, which is given for a collection of harmonic oscillators in thermal equilibrium with the bath of temperature *T* by the well-known relationship:
2

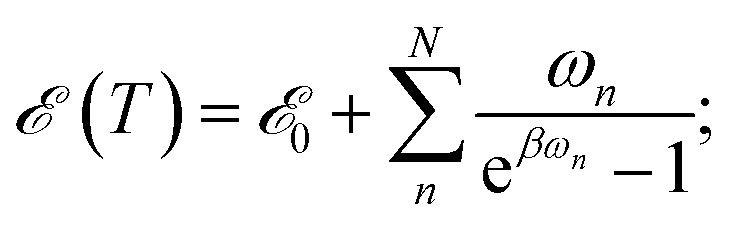

here, *ω*_*n*_ is the frequency of the *n*-th normal mode out of *N* in total (we set *ħ* = 1 throughout this paper), *β* is the inverse temperature and ℰ_0_ formally denotes the zero-point energy. Eqn (2) provides the link between the energy received by the intra-molecular vibrations from the electronic subsystem and the internal molecular temperature. Namely, let us consider the energy of the electronic subsystem only, *ε*_el_, and the vibrational subsystem of energy ℰ(*T*_∞_), where *T*_∞_ is the constant homogeneous temperature of the bulk solvent. The electronic relaxation will eventually deliver energy *ε*_el_ to the vibrational subsystem, where it is shared among the normal modes as if these modes were in quasi-equilibrium with a thermostat of temperature *T*_loc_: ℰ(*T*_∞_) + *ε*_el_ = ℰ(*T*_loc_). This effectively describes the local molecular heating. We note that in real systems some energy from the electronic subsystem likely relaxes directly to the solvent phonon modes. So realistically, under such “leaky” conditions, ℰ(*T*_∞_) + *ε*_el_ ≥ ℰ(*T*_loc_) should be expected. In this work such leaks are disregarded partially due to technical complications of evaluating the size of the effect, partially expecting that in larger molecules such contribution would be diminishing.

In the case of a vibronic system, the energy released during IVR also contributes to the local heating. We assume that the amount of energy dissipated by the vibronic subsystem gets instantaneously spread among the remaining modes thus establishing the Boltzmann quasi-equilibrium and the local temperature.^47^ By this we also implicitly assume that the thermalization of the non-optical intramolecular modes is not hindered by any localization effects.^4^,^48^ The evolution of the local temperature is governed both by IC and IVR and by the heat exchange between the molecule and the solvent, namely VC. The latter process leads to the equilibration with the bulk solvent. In the following we will review the respective in-coming and out-going terms of the equation for the local temperature. We note that typically the build-up of the local temperature is ignored,^21^,^29^ and it is assumed that the starting point of evolution is ℰ(*T*_0_) = ℰ(*T*_∞_) + *ε*, where *ε* is either equal to the energy difference between energy delivered by the pumping laser, *ε*_pump_, and the closest excited state or even *ε* ≈ *ε*_pump_. Such a condition, however, implies instantaneous IC and IVR. Here, we seek to bridge the gap between the infinitesimal and finite IC + IVR in the calculation of temperature evolution.

In order to quantify the increase of the local temperature, we need to keep track of the energy stored in the vibronic subsystem at any given time, *ε*_el_(*t*), as well as the energy delivered by the pumping laser by that time, *ε*_pump_(*t*). The difference between the pumped-in energy and the stored energy is transferred to the vibrational subsystem. Thus, we assume that at every time-step *δt* the change of intramolecular vibrational energy equals

3 *δ*ℰ = [*ε*_pump_(*t* + *δt*) – *ε*_el_(*t* + *δt*)] – [*ε*_pump_(*t*) – *ε*_el_(*t*)].

Alternatively, *δ*ℰ can be viewed as the difference of the energy received (*ε*_pump_(*t* + *δt*) – *ε*_pump_(*t*)); vanishes outside the pumping pulse) and lost (*ε*_el_(*t* + *δt*) – *ε*_el_(*t*)) by the vibronic system during time-step *δt*. Eqn (2) can then be used to obtain the inverse dependence, *T*(ℰ), and to calculate the increase of local temperature *δ*_ℰ_*T* per time-step *δt*. Again, we note that if we included the solvent phonon modes, possibly contributing to the spectral densities of the previous subsection, the local temperature would be lower due to larger number of participating degrees of freedom.

The populations *n*_*a*_^*i*^(*t*) can be used to evaluate the *δ*ℰ in eqn (3) as follows. The total energy of the vibronic subsystem *ε*_el_(*t*) is given by
4

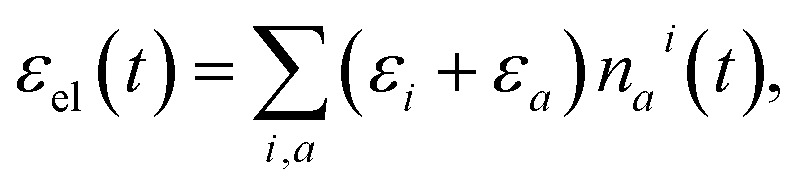

where *ε*_*a*_ = *ω*_1_*a*_1_ + … + *ω*_*K*_*a*_*K*_. We can also keep track of the energy actually delivered to the vibronic subsystem by the pumping pulse, which typically excites a limited number of vibronic levels *a*′ on manifold(s) *i*′. This amounts to
5

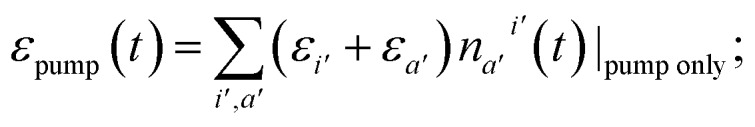

here, “pump only” signifies that populations *n*_*a*′_^*i*′^(*t*) are the solutions to the equations of motion with only the pumping term and no subsequent relaxation present. Thus, *ε*_pump_(*t*) starts from zero and acquires a stationary value corresponding to the pumping frequency within the duration of the pumping time.

Should the molecule be isolated from the solvent, its local temperature would rise on the time scale corresponding to its excitation lifetime to the maximal value, *T*_max_, determined by the laser pulse and the distribution function eqn (2). However, in real situations the molecule is in contact either with the solvent molecules or some kind of embedding matrix. In this paper we are considering the former situation, although the latter is of considerable biological interest in the case of pigments in proteins. The molecular cooling of solute molecules has been investigated to a different degree in several studies.^12^,^29^ Here, we employ an approach analogous to the one given by Kovalenko *et al.*, where the solvent is partitioned into the FSS and the bulk.^12^ The local temperature, from hereon denoted as *T*, decreases due to the heat transfer to the FSS of the temperature *T*_S_, which in turn cools down by heat diffusion to the remainder of the solvent of temperature *T*_∞_. Such a process can be described, along with the earlier prescription for heating, by coupled differential equations:
6

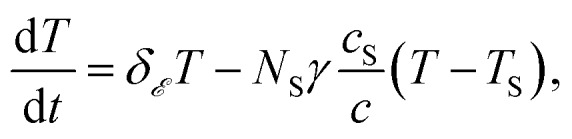



7

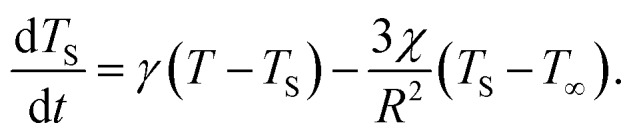

Here, *N*_S_ is the number of solvent molecules within the FSS of radius *R*, *γ* is an empirical coupling strength, *c*_S_/*c* is the heat capacity of the solvent/solute, and *χ* is the diffusivity of the solvent. The second term on the r.h.s. of eqn (7) describes the diffusive cooling of a sphere of radius *R*, and it is derived starting from the Fourier equation.^12^ The parameters entering eqn (6) are of very different nature: while *γ* is purely phenomenological and depends on the specific solvent–solute interactions, the diffusivity can be evaluated from the other well known solvent parameters, as *χ* = *κM*/*Cρ*, where *κ*, *M*, *C* and *ρ* are the thermal conductivity, molecular mass, heat capacity and density, respectively. We denote the solute-to-solvent heat transfer time as *τ*_ss_ = (*N*_S_*γ*)^–1^ and the FSS cooling, or “shell cooling”, time as *τ*_SC_ = *R*^2^/3*χ*. Taken together with the equations governing the vibronic population evolution, eqn (6) and (7) provide a method for the description of local heating and cooling.

### 2.3 Probing dynamics by transient absorption spectroscopy

We inspect the dynamics of the photoexcited system by means of TA spectroscopy.^49^ The time- and frequency-resolved TA spectrum *A*(*ω*, *t*) can be given as the following decomposition:

8 *A*(*ω*, *t*) = *A*_A_(*ω*, *t*) – *A*_E_(*ω*, *t*) – *A*_A_(*ω*, ∞).

Here, *A*_A_(*ω*, *t*) and *A*_E_(*ω*, *t*) denote all the absorptive (*i.e.*, both ground state and induced absorption; IA) and emissive (stimulated emission; SE) contributions. When the delays between the pumping and probing pulses are larger than the pulse durations, the *A*_A_(*ω*, *t*) and *A*_E_(*ω*, *t*) terms can be given by factorizing them into the temporal and frequency parts (coherent effects are neglected as well).^42^ Particularly, the *A*_A_(*ω*, *t*) is given by:
9

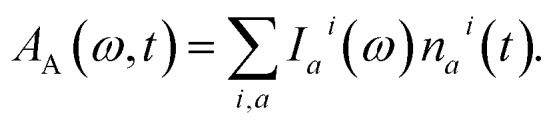




Here, the response in the time domain is given by the populations of the vibronic levels, *n*_*a*_^*i*^(*t*), while the response in frequency domain is described by the spectral shape term *I*_*a*_^*i*^(*ω*):
10

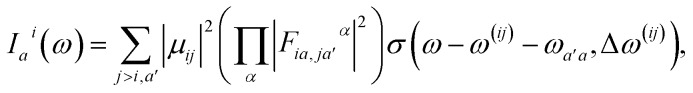

where *μ*_*ij*_ is the electronic transition dipole moment of the *i* → *j* transition, and *σ*(*ω*, Δ*ω*^(*ij*)^) determines the line-shape of the *i* → *j* transition (Gaussian or Lorentzian of FWHM Δ*ω*^(*ij*)^). *ω*^(*ij*)^ = *ε*_*i*_ – *ε*_*j*_ is the 0–0 (purely electronic) energy gap and *ω*_*aa*′_ = *ε*_*a*_ – *ε*_*a*′_ denotes the energy gap between the vibronic states on different manifolds. Similarly, the SE contribution, *A*_E_(*ω*, *t*), is given by the same eqn (9) and (10), except that now 0 < *j* < *i* (transitions to vibronic levels of lower electronic manifolds).

It is noteworthy that in principle the internal temperature manifests in TA spectra in several ways. Firstly, it affects the relaxation rates and thereby also governs the quasi-equilibrium population distribution, once it is established. Secondly, it changes the line-widths of the spectra due to increased participation of the low frequency modes.^50^ The latter effect is incorporated *via* a phenomenological relation:^12^,^51^

11 (Δ*ω*^(*ij*)^(*T*))^2^ = (Δ*ω*^(*ij*)^(*T*_∞_))^2^ + *η*^2^(*T* – *T*_∞_),

which is based on the fact that the line-width of the transition can be partitioned into the temperature-dependent part and the part describing temperature-independent broadening mechanisms (such as static disorder^52^). The broadening parameter *η* is to be obtained from an experiment for a particular molecule and solvent combination.

### 2.4 Protocol for modeling multiple relaxation layers

**Fig. 2:**
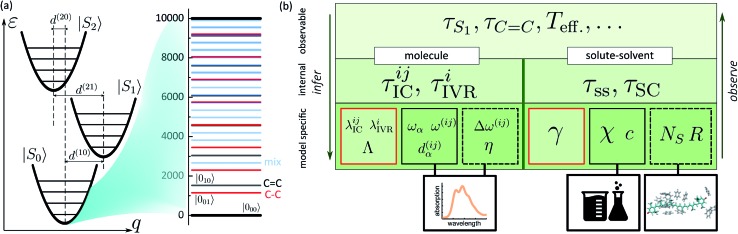
(a) Schematic representation of a three electronic level system with the associated vibronic manifolds. Ground state vibronic manifold of the two high-frequency carbon–carbon stretching modes is explicitly shown on the right. (b) Connection between the parameters governing the evolution of the joint molecule-and-solvent system and the possible experimental observables. The bottom tier summarizes the model parameters: molecule/solvent parameters in the solid black frames are mostly model-independent; parameters in the dashed frame depend on a specific model, but can be verified independently; parameters in the red frames are specific to the presented model and can only be inferred in this specific set-up.

In the previous sections we described a general model of a molecule coupled to the environment. Here, we introduce a particular model considered further in the paper and particular procedures used to implement the presented framework. For the ease of discussion, in the following we will refer to the manifold of vibronic levels |*i*_*a*_〉 as a state |S as a state |S_*i*_〉. We will consider three such states, |S. We will consider three such states, |S_0_〉, |S, |S_1_〉 and |S and |S_2_〉, and their vibronic manifolds of two high-frequency modes, hence, , and their vibronic manifolds of two high-frequency modes, hence, *a* = (*a*_1_, *a*_2_). Such modes associated with carbon–carbon double- and single-bond stretching (frequencies at around 1150 and 1520 cm^–1^, respectively) are typical for polyenes and aromatics.^51^ The three states are shown schematically in Fig. 2a along with the explicit manifold of the vibronic levels in the electronic ground state, |S_0_〉..

As described earlier, there are two kinds of relaxation processes in the considered system: the IC transitions between states |S_*i*_〉 and vibrational relaxation within these states, namely IVR. In order to parametrize the relaxation rates, we assume the overdamped Brownian oscillator spectral density and vibrational relaxation within these states, namely IVR. In order to parametrize the relaxation rates, we assume the overdamped Brownian oscillator spectral density^52^ of the form 𝒞_OBO_(*ω*) = 2*λωΛ*/(*ω*^2^ + *Λ*^2^), where *λ* is the reorganization energy and *Λ* is the damping rate. The latter parameter determines the overall shape of the spectral density. For simplicity it is assumed identical for all the processes and assigned a value of (163 fs)^–1^ based on earlier modeling.^42^ The reorganization energies govern the strength of coupling to the bath and are given individually for a specific process in the model. The |S_*i*_〉 → |S → |S_*j*_〉 IC rates are governed by reorganization energies IC rates are governed by reorganization energies *λ*_IC_^*ij*^, while vibrational relaxation within each of the three manifolds is governed by *λ*_IVR_^*i*^ (identical for both modes as stated earlier). Together with more directly experimentally accessible parameters, such as energy gaps *ω*^(*ij*)^, frequencies *ω*_*α*_ and curve displacements *d*_*α*_^(*ij*)^, they determine the time scales of particular vibronic transitions. Let *τ*_IC_^*ij*^ and *τ*_IVR_^*i*^ denote the sets of inverse rates for IC transitions and IVR within the manifolds, accordingly. Depending on the separation of these time scales, they may of may not directly correspond to the experimentally observed lifetimes of the states and vibrations, *e.g. τ*_S_1__ or *τ*_C=C_. The hierarchy structure of model parameters and observables is illustrated in Fig. 2b: the bottom tier represents the parameters dependent on a particular model, such as given here; the middle tier corresponds to the internal parameters of the system that could in principle be obtained from an alternative model; the top tier represents the typical experimental observables, which correspond to the parameters of the middle tier either directly or indirectly, depending, *e.g.*, on the separation of time scales.

Similarly to the molecular evolution, the principles of quantifying the solute–solvent heat exchange are summarized on the right hand side of Fig. 2b scheme. Two important parameters, the mean solvation number *N*_S_, and FSS radius *R*, can be estimated from the MD simulations. This is done in the following way. From an MD trajectory of a single solute molecule in the solvent we calculate the radial distribution function^31^ (RDF), the first minimum of which gives the radius *R*. In the case of large molecules which are spatially distributed to the extent comparable or even larger than *R*, certain approximations need to be invoked. Firstly, the molecule is partitioned into segments. The segmentation is rather arbitrary, but the obvious criterion is to reduce the volume of interest below the sphere of radius *R*. Then both the segments and the solvent molecules are contracted onto their centers of masses. Subsequently, the RDFs are calculated for each individual segment. Lastly, after estimating each individual segment-radius the “solvation volume” of the whole molecule is constructed. The global FSS radius *R*, required in eqn (7), is approximated as the radius of a sphere covering the volume equivalent to the determined solvation volume. Note, that the accuracy of the value of *τ*_SC_ is limited by the deviation of the solvent volume from a sphere for which the Fourier equation was solved. A particular instance of such a procedure is described in the following section. The remaining bulk properties of the solvent, such as the heat diffusivity *χ*, are readily available in chemical datasets. By contrast, the detailed heat exchange between the system and its FSS falls outside the current level of theory, and the corresponding parameter *γ* is determined purely by fitting, as emphasized by the red rectangle in the scheme. From the basic bottom tier parameters we can estimate the solute-to-solvent heat transfer and shell cooling times, *τ*_ss_ and *τ*_SC_, accordingly. However, the real observables of the top tier, such as the induced line-shape changes, also depend on the left part of the scheme, therefore both sides need to be treated self-consistently.

## 3 Results and discussion

### 3.1 Energy relaxation in a vibronic system and its tracing *via* transient absorption

We demonstrate the key features of the developed methodology by applying it to an arbitrary artificial system introduced in the previous subsection. The value of 10 000 cm^–1^ is assumed for both energy gaps *ω*^(10)^ and *ω*^(21)^. We consider pumping the system by a 70 fs pulse centered at 20 000 cm^–1^, which resonantly excites the lowest vibronic level of |S_2_〉, , *i.e.* |2_00_〉, creating population , creating population *n*_0_^2^(*t*). The reorganization energies *λ*^21^, *λ*^1^, and curve displacements *d*^(21)^ are tuned in such a way as to yield both the |S_2_〉 → |S → |S_1_〉 conversion and the vibrational relaxation within the |S conversion and the vibrational relaxation within the |S_1_〉 manifold on sub-picosecond time-scale. The choice of manifold on sub-picosecond time-scale. The choice of *λ*^0^ is dictated by similar values in real systems, as shown in next subsection. Reorganization energy *λ*^10^ provides an effective measure of the |S_1_〉 decay rate. The specific parameter values underlying the studied regimes are given in the ESI. decay rate. The specific parameter values underlying the studied regimes are given in the ESI.† Without loss of generality, the ℰ(*T*) relationship, eqn (2), is taken from a particular molecule analyzed in the following subsection. The local heating parameters *γ*, *N*_S_ are also close to the values of particular molecules reported further, but otherwise completely arbitrary in order to highlight the model features. Namely, the shell consisting of 10 molecules (heat capacity identical to the model system, *c* = *c*_S_) is set to cool on a time scale of *τ*_SC_ = 10 ps, while we set *τ*_ss_ = 2 ps.

**Fig. 3:**
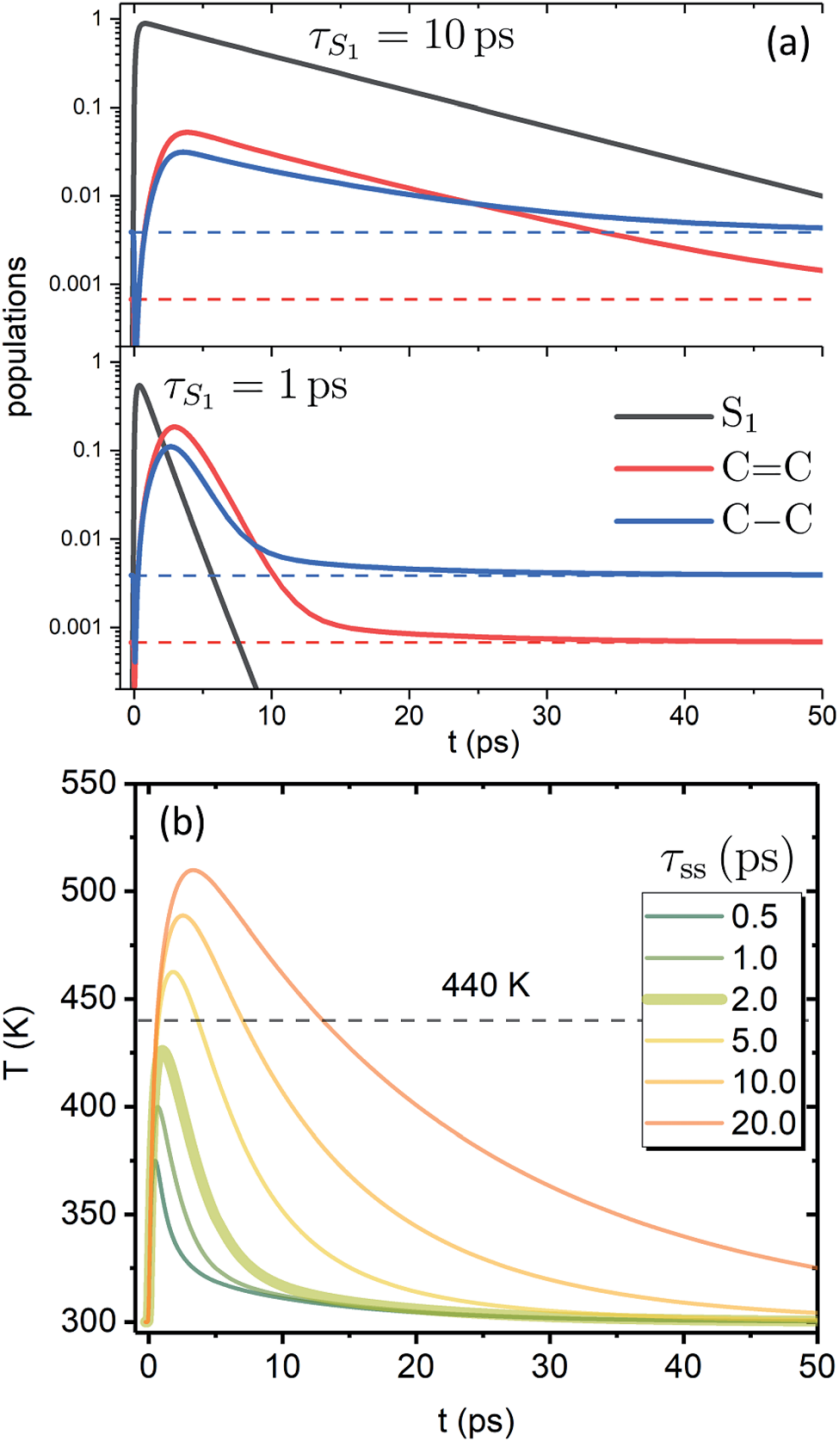
Evolution of the artificial system after optical pumping. (a) Population evolutions. Evolutions of the lowest vibronic levels of C=C and C–C modes in the ground state (*i.e.*, populations of states |0_10_〉 and |0 and |0_01_〉) are shown in red and blue, accordingly. (b) Molecular temperature evolution for different ) are shown in red and blue, accordingly. (b) Molecular temperature evolution for different *τ*_ss_ values (*τ*_S_1__ = 1 ps).

Fig. 3a shows the dependence of the system evolution on the lifetime of |S_1_〉 state, state, *τ*_S_1__. This parameter is evaluated by an exponential fit of the sum of vibronic level populations: 
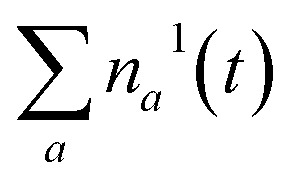
. Evolutions of the lowest vibronic levels of C=C and C–C modes in the ground state, |0_10_〉 and |0 and |0_01_〉 respectively, are also shown as populations respectively, are also shown as populations *n*010 and *n*001. The two panels of Fig. 3a correspond to the cases of *τ*_S_1__ = 10 and 1 ps (top and bottom); the dashed lines show the population equilibrium values at *T* = *T*_∞_ = 300 K. For the time scale comparison, the vibrational relaxation rates based on the chosen *λ*^0^ are (2.34 ps)^–1^ and (1.77 ps)^–1^ for C=C and C–C modes, respectively. Let us firstly look at the *τ*_S_1__ = 10 ps case (top panel). The vibronic ground state levels are initially depopulated by the pumping pulse (note the logarithmic scale), then obtain some transient populations, which slowly relax sustained by the decaying |S_1_〉. The relaxation of the |0. The relaxation of the |0_01_〉 state is clearly not mono-exponential and reaches equilibrium within 50 ps. This suggests that starting with sub-10 ps |S state is clearly not mono-exponential and reaches equilibrium within 50 ps. This suggests that starting with sub-10 ps |S_1_〉 lifetime, the IC ceases to be the rate limiting step and the effects of populated ground state vibronic levels could be observed. Conversely, with extremely short-lived |S lifetime, the IC ceases to be the rate limiting step and the effects of populated ground state vibronic levels could be observed. Conversely, with extremely short-lived |S_1_〉 state, the vibronic states both obtain considerable transient populations and outlast the excited state. They can clearly be seen biexponentially approaching the long-time equilibrium values, the fast exponent corresponding to the IVR times state, the vibronic states both obtain considerable transient populations and outlast the excited state. They can clearly be seen biexponentially approaching the long-time equilibrium values, the fast exponent corresponding to the IVR times *τ*0IVR for each mode, while the slow exponent matches *τ*_SC_.

The corresponding evolution of the molecular temperature for different values of the time scale *τ*_ss_ when *τ*_S_1__ = 1 ps are shown in Fig. 3b; the thickened line corresponds to the *τ*_ss_ value from panel a. For smaller *τ*_ss_ values it is evidently a biexponential process. As can be expected for a solution of two coupled rate equations, the maximal values of the transient species rise/fall with an increase/decrease of *τ*_ss_. Also, with longer *τ*_ss_ the biexponential character of the temperature evolution diminishes. The initial part and the first exponent correspond to the establishment of equilibrium with the FSS, which heats up during this process (not shown) reaching its maximal temperature. The subsequent decay and the second exponent correspond to *τ*_SC_ (the solute and FSS cooling in tandem). The time scale of the initial part and the ratio of maximal temperatures are largely governed by the *γ* parameter, *N*_S_ and the heat capacity ratio. It is noteworthy that according to the distribution function used, the full conversion of 20 000 cm^–1^ excitation energy into internal energy would result in molecular temperature of ∼555 K, while the value of 440 K, shown by the dashed line, corresponds to the conversion of 10 000 cm^–1^, *i.e.* the energy gap covered by the IVR on the |S_1_〉 state. The fact that for shorter state. The fact that for shorter *τ*_ss_ the maximal temperatures do not reach the latter value signifies that most of the temperature build-up is from the |S_2_〉 → |S → |S_1_〉 IC, which is then efficiently dissipated towards the FSS. The fast time scale of this process underlines that a clear distinction between IVR and VC based on time scale alone may not be feasible. In most cases of molecular cooling analysis it is assumed that the formation of local temperature is nearly instantaneous, which is based on the idea that the intramolecular energy redistribution mediated by nonlinear interactions inside the molecule is much faster than the energy transfer from the molecule to its surroundings. Consequently, the temperature equivalent of the deposited excitation energy is considered to be the starting value of local temperature. IC, which is then efficiently dissipated towards the FSS. The fast time scale of this process underlines that a clear distinction between IVR and VC based on time scale alone may not be feasible. In most cases of molecular cooling analysis it is assumed that the formation of local temperature is nearly instantaneous, which is based on the idea that the intramolecular energy redistribution mediated by nonlinear interactions inside the molecule is much faster than the energy transfer from the molecule to its surroundings. Consequently, the temperature equivalent of the deposited excitation energy is considered to be the starting value of local temperature.^21^,^29^,^53^,^54^ Here, we see that due to the details of the actual build-up of the temperature, its maximal values can be expected to be much smaller than the mentioned predictions based on instantaneous heating.

**Fig. 4:**
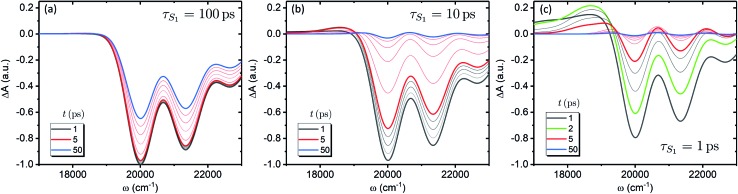
TA spectra of the artificial system. Panels a to c correspond to the |S_1_〉 state lifetimes of 100, 10 and 1 ps, accordingly. All the spectra are normalized to the maximum level of the spectrum at 1 ps for the state lifetimes of 100, 10 and 1 ps, accordingly. All the spectra are normalized to the maximum level of the spectrum at 1 ps for the *τ*_S_1__ = 100 ps parameter set (panel a). The faint gray lines correspond to spectra separated by 1 ps time step, while the faint red lines correspond to 10 ps time step, starting from 10 ps.

**Fig. 5:**
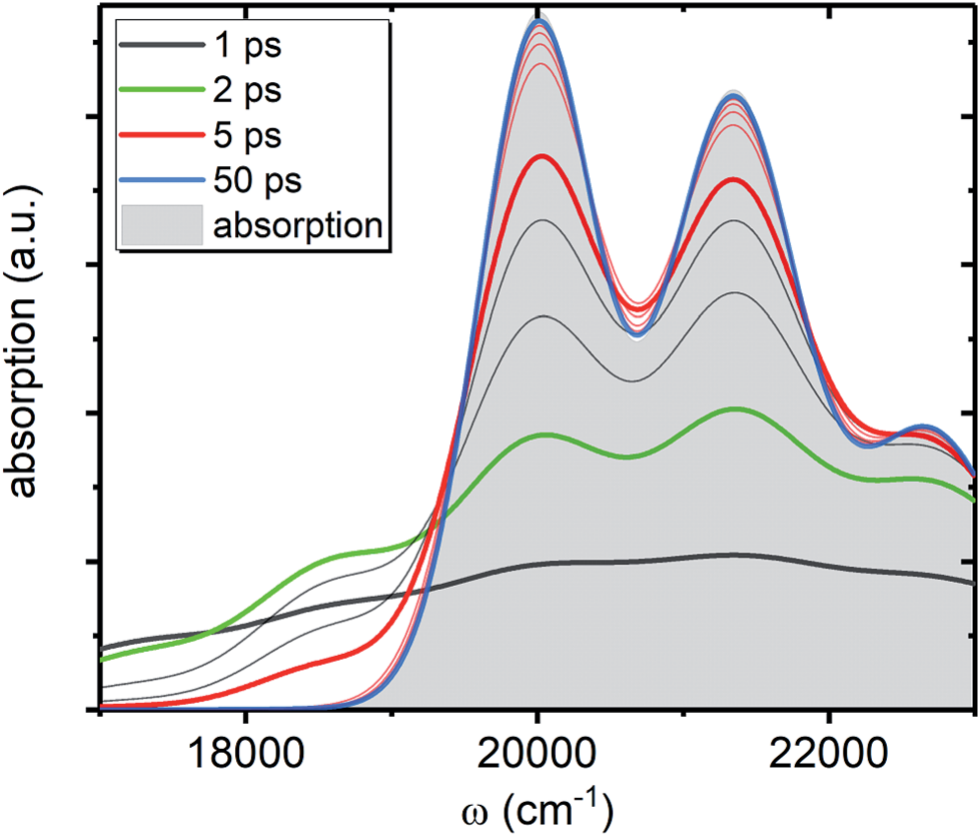
IA component, *A*_A_(*ω*, *t*), from the ground state of the artificial system. All parameters are the same as in Fig. 4c. The shaded contour shows *A*_A_(*ω*, ∞), which is the absorption spectrum of the artificial system. The faint gray lines correspond to spectra separated by 1 ps time step, while the faint red lines correspond to 10 ps time step, starting from 10 ps.

We now focus on how the described dynamics are reflected in terms of experimental observables. In optical experiments, molecular cooling is typically associated with the changes in the red edge of the TA spectra,^12^,^30^ integrated peak intensity^12^,^55^ or fluorescence narrowing.^56^ Here, we focus on the signatures of molecular cooling in the ground state, as reflected in the spectral changes of the ground state bleach (GSB) contribution of the TA spectrum. TA spectra of the artificial system are shown in Fig. 4a–c. Due to the ultrafast (100–200 fs) decay of the |S_2_〉 state and the energetic position of the |S state and the energetic position of the |S_1_〉 state, the frequency window around 20 000 cm state, the frequency window around 20 000 cm^–1^ at times longer than 1 ps is virtually free of contributions from the vibronic levels |2_00_〉 and |1 and |1_*a*_〉. This leaves only the GSB component and potentially some IA from higher ground state vibronic levels. . This leaves only the GSB component and potentially some IA from higher ground state vibronic levels. Fig. 4a shows the simplest case corresponding to *τ*_S_1__ = 100 ps. It features a monotonic decrease (on the time scale of |S_1_〉 lifetime) of the GSB signal, which is just the negative absorption spectrum (conf., shaded area in lifetime) of the GSB signal, which is just the negative absorption spectrum (conf., shaded area in Fig. 5). By contrast, the case of *τ*_S_1__ = 10 ps having qualitatively nearly identical system dynamics, demonstrates entirely new features in the TA spectrum, Fig. 4b. At short times, *t* < *τ*_S_1__, there appears a significant positive feature on the red side of the GSB signal, while at long times, *t* > *τ*_S_1__, alternating positive and negative peaks, reminiscent of the absorption structure, are observed. For the *τ*_S_1__ = 1 ps case, Fig. 4c, the positive feature at ∼19 000 cm^–1^ can now be clearly seen to rise up until 2 ps and then fall. At the same time, the spectrum above 20 000 cm^–1^ is monotonically decaying until the purely negative signal “spills over” into positive values at around 4 ps; see especially the trough at ∼20 800 cm^–1^. To better understand this behavior, let us look at only the IA from the ground vibronic levels, 
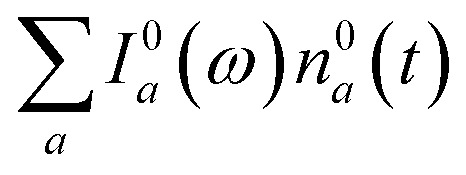
, shown in Fig. 5 along with the absorption spectrum—which technically is 
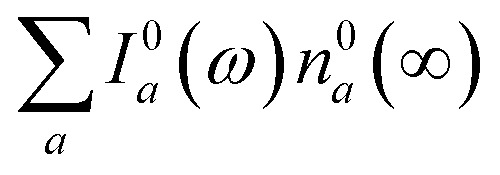
 (shaded contour). The broad and relatively flat IA at 1 ps (black) originates mostly from transitions |0_*a*_1_>1*a*_2_>1_〉 → |2 → |2_*a*_〉. Around 2 ps (green) both vibrational modes acquire their largest transient populations in the first vibronic level, and the IA signal is mostly a composition of |0. Around 2 ps (green) both vibrational modes acquire their largest transient populations in the first vibronic level, and the IA signal is mostly a composition of |0_10_〉, |0, |0_01_〉, |0, |0_00_〉 → |2 → |2_*a*_〉 transitions. Between 5 and 10 ps the IVR is over and quasi-equilibrium is established, transitions. Between 5 and 10 ps the IVR is over and quasi-equilibrium is established, *n*0*a* (10 ps) ≈ *n*0*a* (∞), as can be seen from both the population evolution and the faint red lines of Fig. 5. During the remaining time the only tangible change is due to the spectral line narrowing, which represents the remaining vibrations undergoing VC. The difference between the broad IA and the narrow absorption yields the typical oscillatory structure, which has been observed by Lenzer *et al.* as a difference spectrum of β-carotene solution at different ambient temperatures.^54^

### 3.2 Case study: carotenoids

In this subsection we apply the described strategy and model to a pair of real physical systems. Particularly relevant candidates for such a study are carotenoids, a family of ubiquitous natural pigments participating in vision, photosynthesis and photoprotection.^57^ Their lowest excited state is both short-lived and optically dark, therefore the information about its properties is mostly accessible *via* TA.^18^ Spectral dynamics are typically complex with a number of signals not easily explained in terms of the quantum-chemical electronic structure. In the past decades various signals were being attributed to additional dark states,^37^ which in some cases are now tenuously implicated in key biological functions.^58^,^59^ One such example is the so-called S* signal. It has been reported in TA spectra of carotenoids already in 1995 and was interpreted as a hot ground state signal,^60^ but later “rediscovered” in bacterial light-harvesting pigment–protein complexes and rather interpreted as a separate electronic state.^61^ Although this signal has been argued to represent vibronic transitions from S_1_ and/or hot ground state,^43^,^54^ this interpretation has not yet been unanimously accepted.^62^,^63^ We demonstrate how the presented methodology exhaustively describes the carotenoid TA peculiarities, and elucidates the origin of the S* signal in carotenoids in a conclusive manner.

**Fig. 6:**
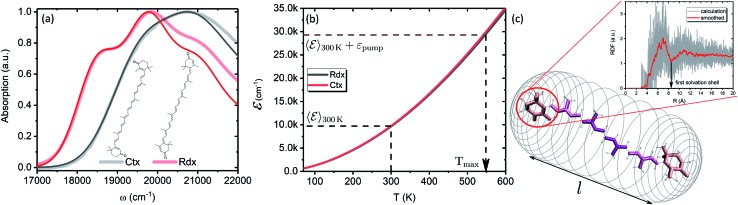
(a) Absorption spectra of Ctx and Rdx in benzene. Thick-faint lines correspond to the measurement, thin lines represent fitting. (b) Internal energy as function of temperature. (c) Schematic representation of the FSS as a cylindrical mesh. Highlighted are the segments of a carotenoid chosen for RDF calculation. The inset shows RDF of an end group of Ctx as obtained from the MD simulation. Red line corresponds to the Savitzky–Golay smoothing for the extraction of FSS radius.

Particularly, we examine two carotenoids: canthaxanthin (Ctx) and rhodoxanthin (Rdx). Their absorption spectra in benzene along with the molecular structures are shown in Fig. 6a. The measured spectra are shown by thick faint lines, thin lines correspond to the model absorption spectra, *I*0*a*(*ω*)*n*0*a*(∞), determined by fitting. For carotenoids *μ*_01_ ≈ 0 because of the inversion and so-called particle-hole/alternancy symmetries,^64^ hence the linear absorption corresponds to the |S_0_〉 → |S → |S_2_〉 transition only. The typical vibronic structure is well pronounced for Rdx, while for Ctx it is obscured by larger line-width. The fitting reproduces the spectra up to the second vibronic peak very well, but then falls below the experimental values. The latter feature is inherent for the harmonic model regardless of the specific implementation, either the “dressed” stick spectrum, as employed here, or the line-shape function transition only. The typical vibronic structure is well pronounced for Rdx, while for Ctx it is obscured by larger line-width. The fitting reproduces the spectra up to the second vibronic peak very well, but then falls below the experimental values. The latter feature is inherent for the harmonic model regardless of the specific implementation, either the “dressed” stick spectrum, as employed here, or the line-shape function *via* cumulant expansion.^65^ The determined parameters are given in full in the ESI.†

In order to asses the energy distribution over the internal degrees of freedom in the carotenoids we perform the quantum chemical calculations of the normal modes. The details of the calculations along with the determined normal modes and their frequencies are provided in the ESI.† The subsequent dependence of the internal energy on the temperature ℰ(*T*), eqn (2), is given in Fig. 6b. Evidently, the dependencies are nearly identical for both carotenoids. At 300 K they both posses ℰ(*T*) ≈ 10 000 cm^–1^, while the laser excitation into their |2_00_〉 levels provides the excess energy of ∼20 000 cm levels provides the excess energy of ∼20 000 cm^–1^ (exact numbers are used in calculations). Should this excess energy be instantaneously converted into the equivalent internal vibrational energy, it would yield the maximal achievable temperature of *T*_max_ = 550 K. This temperature is sometimes assumed as a starting point of temperature evolution,^54^ however, as demonstrated on the previous subsection (we used Ctx ℰ(*T*) for the artificial system), this maximal temperature is unlikely to be achieved due to the dissipation channels which we will consider next. We parametrize the ℰ(*T*) dependencies by analytical functions to obtain the inverted dependencies, *T*(ℰ), for the dynamics calculations.

To parametrize eqn (6) and (7) from the statistics of FSS dynamics, we performed MD simulations of the carotenoids in benzene. The details of MD simulations are given in the ESI.† As mentioned earlier, for molecules as large as Ctx and Rdx (94 and 90 atoms, respectively) some coarse-graining prior to RDF calculations is necessary. We chose to divide the molecules into 6 segments shown in Fig. 6c and contract them onto their mass-weighted centers. In turn, each benzene was coarse-grained onto one particle at the center of mass. The RDFs for all the segments of both carotenoids are largely similar (conf., Fig. S4 in the ESI†), hence, only the RDF for the end group of Ctx is shown in Fig. 6c. Having applied some data smoothing, we can identify the minimum corresponding to the radius of the FSS. Subsequently we assume that the FFS forms a hemisphere-ended cylinder of length equal to the distance between the outermost coarse-grained segments, shown as the mesh in Fig. 6c. We then approximate the radius *R* in eqn (7) as the radius of a sphere equal in volume to the given cylinder (in which case 
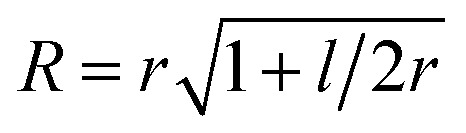
, where *r* is the radius determined from RDF and *l* is the distance between the head groups). Taken with the diffusivity of benzene^29^*χ* = 0.95 (10^–7^ m^2^ s^–1^), this yields shell cooling times of 6.0 ps and 6.45 ps for Rdx and Ctx, respectively.

Additionally, we approximate *N*_S_ as the number of solvent molecules in the vicinity of the solute. The average number of neighboring benzene molecules at distances from 0.5 nm to 0.6 nm increase from 5 to 10 for Rdx and from 6 to 14 for Ctx (see Fig. S5 in the ESI†). We picked the latter values for simulations. We note that while the radius *R* is combined with the fixed parameter *χ* to uniquely yield the time scale *τ*_SC_, the number *N*_S_ is combined with the free parameter *γ* to yield the time scale *τ*_ss_, hence *τ*_ss_ is still a fitting parameter and *N*_S_ merely acts as a constraint to extracting *γ*. The time scale of relaxation to the FSS was taken *τ*_ss_ = (*N*_S_*γ*)^–1^ = 0.5 ps based on similar values determined for stilbene.^12^ As this value could not be taken from an independent measurement or modeling, we attempted to verify its value indirectly. Namely, the extent to which the local temperature manifests in spectra depends on its magnitude, governed by *τ*_ss_ and *τ*_SC_, and the parameter *η via*eqn (11). Measuring the temperature dependence of Rdx absorption spectrum revealed that the value of *η* = 29.76 cm^–1^ K^–1/2^ is a reasonable estimate, which in turn used in the TA calculations together with *τ*_ss_ = 0.5 ps give a good fit. Details of the temperature-dependent measurement are given in the ESI.†

**Fig. 7:**
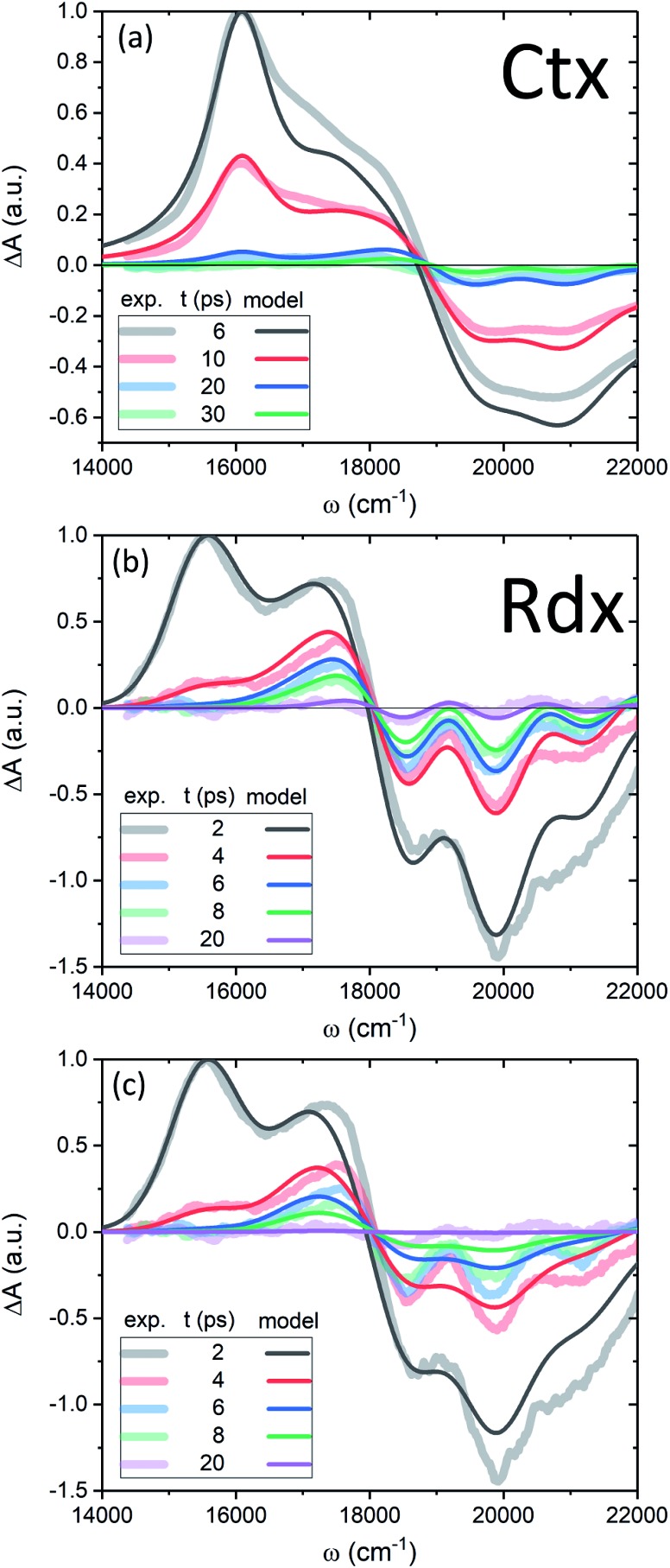
(a, b) TA of Ctx and Rdx, accordingly. Thick-faint lines correspond to measurements, thin lines represent the model fitting. (c) TA of Rdx without the line-width dependence on temperature (*η* = 0).

Now we apply the developed model and the determined parameters to reconstruct the TA spectra of the two carotenoids. Fig. 7 shows the spectra at late times, relevant for the discussion of the molecular cooling, while spectra at early times are provided in Fig. S2 in the ESI† (along with all the parameter values), to demonstrate the consistency of the methodology. Let us consider Ctx, Fig. 7a, as the first example. The measured spectra are shown by thick faint lines, the model corresponds to thin lines. Even though the spectral profile between 16 500 cm^–1^ and 19 000 cm^–1^ shows a mismatch at earlier times, the spectra are clearly decaying on at least two time scales. The IA from |S_1_〉 at 16 000 cm at 16 000 cm^–1^ decays on 4.9 ps time scale, whereas the decay of a signal around 18 000 cm^–1^, termed S*, is visibly slower. An important feature accompanying the emergence of the separate S* signal is the narrowing of GSB.^37^ This narrowing is qualitatively captured by the model very well (conf., Fig. S3 in the ESI†) and is a direct signature of the spectral line-width change. On the one hand, the effect of internal temperature on the line-width is essential to account for the extent of the process, as the presence of “non-thermal” ground state vibronic populations alone is insufficient. On the other hand, evidently these ground state vibronic levels decay at least on the same or slower timescale as the VC, because the signal in the GSB frequency window never crosses the zero line and is purely negative. The molecular temperature is found to decay on the time scale of 10–12 ps (depending on the fitting window). The extracted lifetime of |0_10_〉 population is 8–10 ps. An even longer time constant of |0 population is 8–10 ps. An even longer time constant of |0_10_〉 decay in Ctx (15–20 ps) has been reported from the anti-Stokes Raman measurements. decay in Ctx (15–20 ps) has been reported from the anti-Stokes Raman measurements.^66^ These results provide a clear example of a system in which IVR is at least of the same or possibly even longer time scale as VC.

By contrast, in the Rdx case, both |S_1_〉 relaxation and IVR are considerably faster: relaxation and IVR are considerably faster: *τ*_S_1__ = 0.9 ps, |0_10_〉 lifetime is 3.5 ps. At 20 ps (violet line, lifetime is 3.5 ps. At 20 ps (violet line, Fig. 7b) the oscillatory structure is clearly observable and is identical to the red structure depicted in Fig. 4c. This shows that in Rdx both |S_1_〉 → |S → |S_0_〉 IC and subsequent IVR are rapid enough to reveal the alternating structure typical for VC alone. This also reveals the dual nature of the S* signal at 17 500 cm IC and subsequent IVR are rapid enough to reveal the alternating structure typical for VC alone. This also reveals the dual nature of the S* signal at 17 500 cm^–1^: at 4 ps (red line) this red-detuned satellite of GSB is mostly due to IA from higher vibronic levels on the ground state, at 8 ps (green line) it is a mixed result of higher vibronic levels and elevated molecular temperature, while at the latest times, *e.g.* 20 ps, this positive feature is mostly due to the line-shape differences between the locally “hot” (after pumping) and “cold” (no pumping) molecule. Such interpretation is further strongly supported by comparison of the spectra in Fig. 7b with the modeled TA assuming temperature-independent line-width (by setting *η* = 0 in eqn (11)) shown in Fig. 7c. The most striking difference is the lack of GSB narrowing, demonstrating that the temperature dependence of the line-width is a crucial feature of the underlying model rather than a mere correction. By contrast, the temperature dependence has little effect to the S* signal at times earlier than 20 ps. Even though the inclusion of temperature dependence visibly improves the overall line-shape of the peak, IVR alone is sufficient to explain its presence at these times.

Regarding the long-time evolution, of particular relevance and interest is the FSS radius, *R*, which is the scale parameter crucial for heat conduction, as for any diffusion process. This has been pointed out in the earliest attempts to describe VC.^29^ Regardless, whether the FSS is modeled as a sphere,^12^,^29^ point-source^13^ or a box,^10^ the scale parameter will be decisive in determining the order of *τ*_SC_. The question of the model accuracy beyond that is rather open. For instance, we have disregarded the stability of FSS, which in principle is possible to asses from MD trajectories by estimating the mean dwelling time of a solvent molecule within the FSS. However, the interchange of molecules between FSS and the bulk would clearly reduce *τ*_SC_ by introducing convection as an additional relaxation channel. Hence, we can only expect the model to provide us the upper value of *τ*_SC_. Interestingly, we find that the radii of FSS for various carotenoids/solvents do not differ much (conf., also results for zeaxanthin in THF in Fig. S4c in the ESI†), nor do the diffusivities of various solvents (they vary mostly within 0.8–1.2 × 10^–7^ m^2^ s^–1^).^10^,^29^,^55^ This means that *τ*_SC_ is seemingly rather similar for all carotenoids. Moreover, in various studies of VC the correlation of cooling times and solvent diffusivities has been reported both present^10^,^55^ and absent.^56^ This shows that the decomposition of VC into solute-to-solvent and FSS-to-bulk steps is not straightforward. On the other hand, the *γ* parameter (or, conversely, *τ*_ss_) for a given combination of solute and solvent is variable, yet there is no apparent single method to establish an exact microscopic dependence. For some large organic molecules the transfer to solute time is estimated to be of the order of 10 ps.^8^,^36^ Taking all this into account, it could very well be that the reported variation of S* signal lifetimes from picoseconds^19^ to tens of picoseconds^67^ can be due to the latter process.

As mentioned earlier, the current *γ* parameter is unknown from any direct complementary measurement or model. Therefore we complemented the TA experiment with temperature dependence of the ground state absorption spectra, as pointed out by Lenzer *et al.* The amplitude of the oscillatory structure provides a rather well established connection between *γ* and *η* parameters. The robustness of the model with respect to the variation of *τ*_SC_, *τ*_ss_ and *N*_S_ is demonstrated in Fig. S6 in the ESI,† where we show that the determined values are optimal for the overall fitness of the model. The possible obstacles for relating the current *η* to the change of line-width of absorption spectrum with varied temperature Δ*ω*^(*ij*)^(*T*), apart from the physical limitations (the thermal degradation of the sample), are two-fold. Firstly, uniformly heating the solution also changes some purely solvent properties, such as the refractive index and density of the solvent, leading to the peak shift and change in amplitude. Reversing these effects by data analysis might as well affect the retrieved Δ*ω*^(*ij*)^(*T*) dependence. Secondly, the premises of the two experiments are rather different, and the question whether the temperatures of hot and cold bulk solvent yield identical Δ*ω*^(*ij*)^(*T*) with respect to the molecular temperature alone remains open. Therefore we consider the uniform heating experiments only as complimentary means to constrain *η* and, subsequently, *γ* parameters. It would essentially be intriguing to obtain direct estimates of *γ* from non-equilibrium MD simulations.^36^

## 4 Conclusions and outlook

In this study the explicit treatment of IVR is used to bridge the initial act of photoexcitation of a molecule and the eventual VC mediated by a solvation shell. We specifically analyzed the interplay of the characteristic time scales: *τ*_S_1__ (lifetime of the lowest excited state of the solute), *τ*_C=C_ (lifetimes of particular vibrational modes), *τ*_ss_ and *τ*_SC_ (solute-to-FSS and FSS-to-bulk heat transfer times, respectively). Particular combinations of these time scales determine which effects “ring out” in the observed TA spectra. When *τ*_S_1__ ceases being the limiting rate, the GSB signal is attributed to the hot ground state. Yet, one can evidently discern two types of “hot” state: associated with either the non-equilibrium distribution of vibronic populations or with an elevated local temperature (quasi-equilibrium of all the molecular vibrations). Both effects are sources of change in the GSB signal: IVR leads to the overall decay of the signal and its narrowing due to the vanishing vibronic IA contributions (Fig. 5), while the local temperature manifests *via* the line-width Δ*ω*^(*ij*)^(*T*). If *τ*_C=C_ and *τ*_ss_/*τ*_SC_ are comparable, then both the vibrational populations and the local temperature determine the appearance of GSB. Specifically, a positive shoulder, red-detuned with respect to the ground state absorption, readily stems from non-equilibrium vibronic populations (conf., Fig. 5 and 7c), however, it is enhanced by the thermal dependence of the line-width. On the other hand, given a further separation of timescales, *τ*_C=C_ < *τ*_ss_/*τ*_SC_, we have demonstrated—both in experiment and in the model—the emergence of GSB as alternating positive and negative values (conf., Fig. 4c and 7b). This oscillatory structure is a telltale sign of the elevated local temperature and immediately informs that IVR is mostly finished. Finally, our model shows that the initial build-up of the local temperature due to time dependence of IVR cannot be disregarded and the conjecture of ultra-fast IVR should be treated with caution.^56^ The details of the initial heating determine the maximal achievable temperature (conf., Fig. 3b), which in turn is crucial in calibrating any internal “thermometer”, a physical observable indicative of the local temperature. The presented model could be nicely complemented (and tested) by measurements of the solvent temperature, and indeed such type of measurements for certain systems are already reported.^68^,^69^

The applicability of the theoretical analysis was demonstrate by inspecting photo- and temperature dynamics in carotenoids, Ctx and Rdx.^70^ The well resolved spectra and a very short-lived S_1_ state (∼1 ps) of Rdx lead to the observation of interplay between the vibronic and thermal effects. After S_1_ signal is gone, substantial populations of levels |0_*a*_1_>0*a*_2_>0_〉 are clearly present, as the signal in the GSB region is purely negative. By contrast, at the latest times, the oscillatory structure emerges, which is associated with elevated local temperature of the molecule relaxed to the lowest ground state vibronic level |0 are clearly present, as the signal in the GSB region is purely negative. By contrast, at the latest times, the oscillatory structure emerges, which is associated with elevated local temperature of the molecule relaxed to the lowest ground state vibronic level |0_00_〉. Conversely, Ctx never demonstrates the oscillatory spectral features. The main reason for this is the slower IVR, thus, Ctx is an example of a system where IVR and VC are concomitant rather than sequential. The obtained results also illustrate the dual origin of the S* signal as absorption from the hot ground state: before IVR is over, S* is a “hot” state in a dual sense of both non-equilibrium vibronic populations and increased local temperature; at times exceeding IVR, the S* signal indicates increased local temperature only. The complicated nature of the multiple relaxation processes of similar time scales explains the multi-exponential decay of S* in longer-chain carotenoids.. Conversely, Ctx never demonstrates the oscillatory spectral features. The main reason for this is the slower IVR, thus, Ctx is an example of a system where IVR and VC are concomitant rather than sequential. The obtained results also illustrate the dual origin of the S* signal as absorption from the hot ground state: before IVR is over, S* is a “hot” state in a dual sense of both non-equilibrium vibronic populations and increased local temperature; at times exceeding IVR, the S* signal indicates increased local temperature only. The complicated nature of the multiple relaxation processes of similar time scales explains the multi-exponential decay of S* in longer-chain carotenoids.^19^ We conclude that the presented research ultimately corroborates the interpretation of the S* signal in long carotenoids in terms of a hot ground state.^42^,^43^,^54^,^60^,^71^

In summary, our model unifies energy relaxation processes within a molecule and into the surrounding medium, and reveals specific features of TA spectra, characteristic to IVR and VC processes. Most of the model parameters can be obtained by complementary measurements, therefore the model is highly consistent. Its predictive power is demonstrated by disentangling various relaxation pathways in molecules as large and complicated as carotenoids, where the coincidental signal lifetimes lead to the appearance of the absorptive features of the hot molecule in disguise as an excited electronic state. Hence, carotenoids are a suitable candidate “thermometer” for testing specific theories of solute–solvent interactions and intermolecular vibrational energy transfer. Such studies are important in understanding the cooling of pigment–protein complexes^13^ or the possible roles of hot states in the biological protein function. For example, a recent suggestion that the S* state triggers the photoactivation of the orange carotenoid protein (OCP) is a striking example.^59^ The reported lifetime of the S* state in OCP is much longer than that of the S_1_ state and is in line with our assignment to the hot ground state. Thus, the energy of a hot ground state of the carotenoid bound to OCP could be exploited to break the hydrogen bonds and initiate the OCP photocycle. Moreover, the role of solute-to-solvent energy transfer and local heating effects in the chemical reactivity and selectivity has been brought into question,^36^ as it is becoming evident that in some cases the inclusion of non-instantaneous thermalization might be necessary to adequately capture the chemistry of reactive intermediates.^5^,^6^

## Conflicts of interest

There are no conflicts to declare.

## Supplementary Material

Supplementary information
