# Supplementary material for: The full dynamics of energy relaxation in large organic molecules: from photo-excitation to solvent heating
Source: Chem Sci. 2019 Apr 2;10(18):4792–804. doi: 10.1039/c9sc00410f (PMC6521204; doi:10.1039/c9sc00410f)
Supplement: Supplementary file 1 [file SC-010-C9SC00410F-s001.pdf]

Electronic Supplementary Material (ESI) for Chemical Science.
This journal is © The Royal Society of Chemistry 2019

Supplementary Information to the Article "The full dynamics of energy relaxation in large organic molecules: from photo-excitation to solvent heating"

Vytautas Balevičius Jr.,[a] Tiejun Wei,[a] Devis Di Tommaso,[a] Darius Abramavicius,[b] Jürgen Hauer[c,d], Tomas Polívka[e] and Christopher D. P. Duffy*[a]

1 Theory and model

1.1 Equations of Motion

In this section we provide the equations of motion for the system described by the vibronic Hamiltonian as obtained from the initial Hamiltonian given in the main text:

$$\begin{aligned} H \quad = \quad & \sum_{i,a} |i_a\rangle\langle i_a|(\varepsilon_i+\varepsilon_a) \\ & + \sum_{\kappa,i,a} c_{i\kappa}x_\kappa\sqrt{a_\alpha+1}\left(|i_a\rangle\langle i_{(a_\alpha+1)}|+|i_{(a_\alpha+1)}\rangle\langle i_a|\right) \\ & + \sum_{\kappa,i,a,a'} f_{i\kappa}x_\kappa\left(\left(\prod_\alpha {}^i\langle a_\alpha|a'_\alpha\rangle^{i+1}\right)|i_a\rangle\langle i+1_{a'}|+\left(\prod_\alpha {}^{i+1}\langle a_\alpha|a'_\alpha\rangle^i\right)|i+1_a\rangle\langle i_{a'}|\right) \\ & + \sum_\kappa\left(\frac{p_\kappa^2}{2m_\kappa}+\frac{m_\kappa\omega_\kappa^2}{2}x_\kappa^2\right). \end{aligned} \tag{1}$$

Here, the vibronic basis of states $|i_a\rangle = |i\rangle|a_1\rangle^i\ldots|a_N\rangle^i$ is used, where $|a_\alpha\rangle^i$ denotes the wave-function of the α-th harmonic oscillator on the i-th electronic state, and the entries in the tuple $a=(a_1,\ldots,a_\alpha,\ldots,a_N)$ indicate the number of quanta of the α mode with energy spacing ω_α within the associated electronic state. We defined an auxiliary vibrational energy matrix $\varepsilon_a=\omega_1a_1+\ldots+\omega_Na_N$, and $(a_\alpha+1)$ on the second line denotes a tuple that differs from a by the α-th entry, which is increased by one. On the third line $F^\alpha_{ia,ja'} = {}^i\langle a_\alpha|a'_\alpha\rangle^j$ denotes the Franck–Condon factors. Broadly, the first term represents the static energies of the vibronic basis states, the second term generates transitions within the manifold of vibrational levels associated with the same electronic state (purely vibrational relaxation), while the third term generates transitions between vibrational manifolds belonging to different electronic states (internal conversion, IC). The final term represents the modes of the bath.

The equations of motion for the populations of the vibronic states $|i_a\rangle$, n^i_a, are derived from the quantum Liouville equation, by the second-order perturbation theory with respect to the system–bath coupling, as described in Ref. [1]. Essentially, they are Markovian rate equations of the following form:

$$\frac{\mathrm{d}}{\mathrm{d}t}n^i_a = \left(\frac{\mathrm{d}}{\mathrm{d}t}n^i_a\right)_f + \left(\frac{\mathrm{d}}{\mathrm{d}t}n^i_a\right)_c, \tag{2}$$

where the first term describes the internal conversion dynamics and the second one describes the vibrational relaxation:

$$\begin{aligned} \left(\frac{\mathrm{d}}{\mathrm{d}t}n^i_a\right)_f \quad = \quad & \sum_{a'}\left(\prod_\alpha\left|F^\alpha_{ia,i+1a'}\right|^2\right)\left[-k^{i+1i}_{a',a}n^i_a+k^{ii+1}_{a',a}n^{i+1}_{a'}\right] \\ & + \sum_{a'}\left(\prod_\alpha\left|F^\alpha_{ia,i-1a'}\right|^2\right)\left[-k^{i-1i}_{a,a'}n^i_a+k^{ii-1}_{a,a'}n^{i-1}_{a'}\right]. \end{aligned} \tag{3}$$

* E-mail: c.duffy@qmul.ac.uk
[a] School of Chemical and Biological Sciences, Queen Mary University of London, Mile End Road, London E1 4NS, UK.
[b] Institute of Chemical Physics, Vilnius University, Sauletekio av. 9, LT-10222, Vilnius, Lithuania.
[c] Fakultät für Chemie, Technical University of Munich, Lichtenbergstraße 4 D-85748 Garching, Germany.
[d] Photonics Institute, TU Wien, Gußhausstraße 27, 1040 Vienna, Austria.
[e] Institute of Physics and Biophysics, Faculty of Science, University of South Bohemia, Branišovská 1760, 37005 České Budějovice, Czech Republic.

$$\left(\frac{\mathrm{d}}{\mathrm{d}t}n_a^i\right)_c = -\sum_\alpha \left(a_\alpha k^+(\omega_\alpha) + (a_\alpha+1)k^-(\omega_\alpha)\right) n_a^i + \sum_\alpha \left((a_\alpha+1)k^+(\omega_\alpha)n^i_{(a_\alpha+1)} + a_\alpha k^-(\omega_\alpha) n^i_{(a_\alpha-1)}\right). \quad (4)$$

Here, we have defined two kinds of rate constants: those between the vibrational levels of different electronic manifolds, k^{ji}, and those between the neighboring vibrational levels of the same electronic manifold only, $k^{\pm}$. The latter are explicitly shown as downhill rates $k^+(\varepsilon)$ or the complementary uphill rates $k^-(\varepsilon)$, corresponding to the energy gap ε. These rates are obtained from the Fourier transform of the bath correlation function[1], which is related to the corresponding spectral densities $\mathscr{C}_i^c(\varepsilon)$ and $\mathscr{C}_{ij}^f(\varepsilon)$ as

$$C(\varepsilon) = \mathscr{C}(\varepsilon)\left(\coth\left(\frac{\beta\varepsilon}{2}\right)+1\right), \quad (5)$$

where $\beta = (k_\mathrm{B}T)^{-1}$ is the inverse temperature. The downhill/uphill rate is then $k^{\pm}(\omega_\alpha) = C_c(\pm|\omega_\alpha|)$. The rates k^{ij} can be given by firstly defining the energy gap between the vibronic states: $\Delta^{ij}_{a,a'} = \omega^{(ij)} + \omega_{aa'}$, where $\omega^{(ij)} = \varepsilon_i - \varepsilon_j$ is the purely electronic energy gap and $\omega_{aa'} = \varepsilon_a - \varepsilon_{a'}$ denotes the energy gap between the vibronic states on different electronic manifolds. We then have the rates as $k^{i>j}_{a,a'} = C_f(\Delta^{ij}_{a,a'})$ and $k^{i<j}_{a,a'} = C_f(-\Delta^{ji}_{a,a'})$. We note that by the virtue of Eq. 5 the presented model has readily inbuilt thermodynamics.

In order to describe the dynamics of the given vibronic system after photo-excitation we assume that the system is initially in the thermally populated vibronic ground state, i.e.,

$$n_a^0(\infty) = \mathrm{e}^{-\beta\varepsilon_a}\prod_\alpha\left(1-\mathrm{e}^{\beta\omega_\alpha}\right), \quad (6)$$

and append to the given equations the following pumping terms:

$$\left(\frac{\mathrm{d}}{\mathrm{d}t}n_y^x\right)_\mathrm{pump} = \Gamma(t,\tau_\mathrm{p}); \quad (7)$$

$$\left(\frac{\mathrm{d}}{\mathrm{d}t}n_a^0\right)_\mathrm{pump} = -\Gamma(t,\tau_\mathrm{p}),$$

where n_y^x is the population of resonantly selected vibronic state(s) and $\Gamma(t,\tau_\mathrm{p})$ is the Gaussian function (τ_p is the pump pulse duration).

2 Carotenoid spectra

2.1 Experimental details

Rhodoxanthin (Rdx) and canthaxanthin (Ctx) were purchased from Carotenature (Basel, Switzerland). Absorption and TA experiments were performed at room temperature.

Temperature dependence. Temperature dependent absorption spectra of Rdx were measured in a Shimadzu UV-2600 spectrophotometer equipped with a temperature controlled cell holder TCC-240A (Shimadzu, Japan). The cell holder is an electronically controlled Peltier unit, temperature is regulated for both the sample and the reference cell. The instrument slit width was set to 2 nm. During the experiment, temperature was allowed to stabilize in the cell holder, and a 10-minute equilibration time before each measurement was used to ensure that the sample temperature was well stabilized prior to measurement of absorption spectrum at a particular temperature. Absorption spectra were measured in a 1 cm path length quartz cell.

Femtosecond transient absorption spectroscopy. Femtosecond TA data were recorded by a femtosecond spectrometer based on Integra-i (Quantronix) amplified laser system producing ∼130 fs pulses with 795 nm central wavelength at a repetition rate of 1 kHz. The amplifier output was divided into two paths. The first one was directed to the optical parametric amplifier TOPAS (Light Conversion) to generate tuneable excitation pulses. The second one to produce white-light continuum probe pulses in a 2 mm sapphire plate. The probe and the reference beams were brought to the slit of a spectrograph where it was dispersed onto a double photodiode array allowing measurements of transient spectra in a spectral window of ∼240 nm. In all measurements the mutual polarization of pump and probe beams was set to the magic angle (54.7^o) by placing a polarization rotator in the pump beam. All measurements were carried out in a rotational cuvette consisting of two 1 mm quartz windows separated by a 1 mm Teflon spacer.

2.2 Temperature dependence of Rdx absorption spectrum

The absorption spectra shown in the main text were fitted first obtaining the values of parameters $\omega^{(02)}$, $\Delta\omega^{(02)}$, $d^{(02)}_{1,2}$ as given in the Table S1. The temperature dependent spectra of Rdx are shown in Fig. S1 (left panel). The spectra are normalized to the maximum peak, which eliminates the amplitude variation due to the change of the properties of the solvent, as discussed by Lenzer *et al.*[2]. The

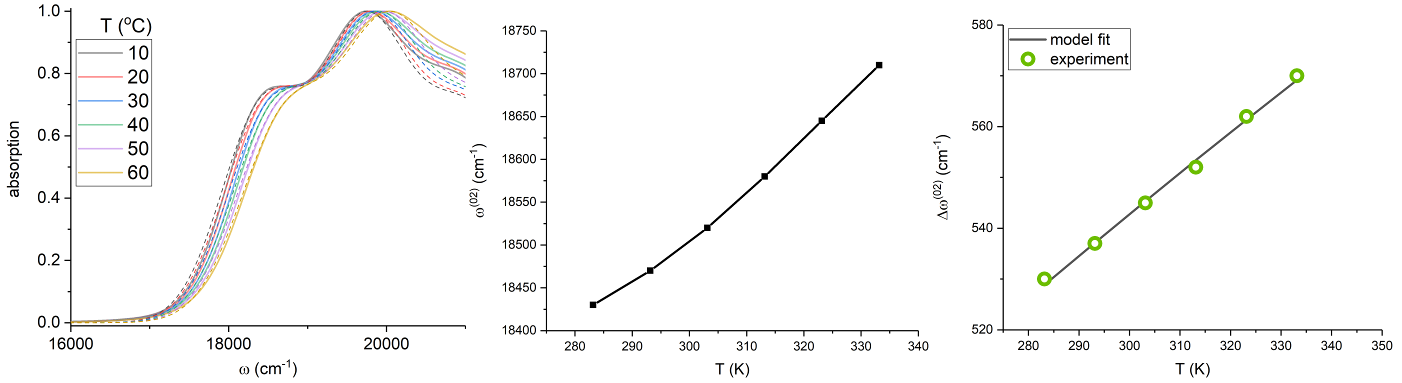

Figure S1 Temperature dependence of Rdx absorption spectrum. Panels left to right: spectral fits; thermal peak-shift; width dependence on temperature (green circles — results from spectral fits, black line — global fit of the dependence).

spectra were then fitted using the same procedure varying only the 0-0 frequency, which shifts due to temperature dependence of the refractive index of the solute[2], and the width. The fitting results are shown by dashed lines in the left panel of Fig. S1. For the sake of completeness, the extracted peak shift is given in the central panel of Fig. S1. Width dependence on temperature is shown in the rightmost panel: the width fitting from the experimental data results are shown by the circles; the line represents the global fitting of the results by the model $\left(\Delta\omega^{(ij)}(T)\right)^2 = \left(\Delta\omega^{(ij)}(T_\infty)\right)^2 + \eta^2(T - T_\infty)$. The determined parameter values are $\Delta\omega^{(ij)}(T_\infty) = 170\,\mathrm{cm}^{-1}$ and $\eta = 29.76\,\mathrm{cm}^{-1}\mathrm{K}^{-1/2}$ yields the parametrization closest to the experiment in the given temperature window.

2.3 Carotenoid parameters from TA

Modeling and fitting carotenoid TA spectra requires determining the remaining molecular parameters (in addition to the readily-known values of $\omega^{(02)}$, $\Delta\omega^{(02)}$, $d_{1,2}^{(02)}$ from the absorption fitting). They can be grouped into "line-shape" parameters and "dynamic" parameters, as emphasized by black and blue notation in Table S1, respectively. The former group consists of the $\omega^{(1n)}$, $\Delta\omega^{(1n)}$, $d_{1,2}^{(1n)}$ to describe the IA from the $|S_1\rangle$ state, as well as the ratio of the GSB/IA amplitudes, $|\mu_{02}|^2/|\mu_{1n}|^2$; a normalization condition $|\mu_{1n}|^2 = 1$ is used. The Stokes shift for $|S_2\rangle$-to-$|S_0\rangle$ transition, $\delta\omega_{\mathrm{St.}}^{(02)}$, is needed to properly position the short-lived stimulated emission (SE) contribution; it is given in the last line of Table S1. The reorganization energies $\lambda_{\mathrm{IC}}^{ij}$, $\lambda_{\mathrm{IVR}}^{i}$ and displacements $d_{1,2}^{(01)}$, $d_{1,2}^{(12)}$ comprise the second group of parameters. While the reorganization energies $\lambda_{\mathrm{IC}}^{ij}$ mostly govern only the decay of the corresponding signal components, strictly speaking, $\lambda_{\mathrm{IVR}}^{i}$ and the displacements also influence the overall line-shape (as opposed to just individual vibronic transition $\sigma(\omega, \Delta\omega^{(ij)})$). Namely, $\lambda_{\mathrm{IVR}}^{i}$ govern the narrowing of the spectra due to vibrational relaxation. The extent of such narrowing in turn depends on the "dynamic" displacements $d_{1,2}^{(01)}$, $d_{1,2}^{(12)}$ (conf., the "static" displacements $d_{1,2}^{(1n)}$, $d_{1,2}^{(02)}$). They influence the global line-shape by determining which vibronic levels are the primary recipients of the excitation during IC (the larger the displacement — the higher vibronic levels get populated upon the IC first; the higher vibronic levels involved in an optical transition — the broader overall IA signal from that vibronic manifold). Some of the dynamic parameters can be verified experimentally, e.g. $\omega^{(12)}$, $d_{1,2}^{(12)}$ can be determined from the TA in the near infrared[3]. Here, we only resort to the typical values, also assuming $\omega^{(01)} + \omega^{(12)} = \omega^{(02)}$, and adjust the reorganization energies accordingly. The long-time fitting results are shown in the main text. Here, we additionally provide the early/intermediate times, Fig. S2, to demonstrate that the method uniformly captures all times from tens of femtoseconds (to tens of picoseconds in the main text).

2.4 GSB narrowing of Ctx: model vs experiment.

Although quantitative agreement between the model and the experiment is poorer for Ctx, it is possible to show extremely good qualitative agreement in the following way. Normalizing the experimental TA spectra to the maximum of the IA from the $|S_1\rangle$ state reveals two trends as demonstrated in the top panel of Fig. S3. Firstly, an increase of positive signal around $18000\,\mathrm{cm}^{-1}$is observed in time; this is a good technique to demonstrate the existence of S^* signal when it is not well separated from the $|S_1\rangle$ lifetime (which is the case for, e.g., Rdx). Secondly, the associated narrowing of GSB is clearly taking place. It has been observed earlier that such GSB narrowing accompanies the presence of S^* signal. In this work we provide the underlying details of such relationship. This can be seen from the bottom panel demonstrating the corresponding modeling results.

2.5 Quantum-chemical calculation details

The normal modes of carotenoids for the distribution function were calculated after vacuum optimization using the Gaussian 09 package[4] with a B3LYP exchange correlation functional with the 6-311g(2d,p) Pople basis set. Normal mode frequencies for Rdx and Ctx

Table S1 Molecular input parameters for the modeling of the TA speactra.

parameter	artificial system	Ctx	Rdx
$\omega^{(01)}$ (cm^{-1})	10000	13000	11970
$\omega^{(12)}$ (cm^{-1})	10000	6550	6550
$\omega^{(02)}$ (cm^{-1})	20000	19550	18520
$\omega^{(1n)}$ (cm^{-1})	n.a.	16080	15530
λ_{IC}^{21} (cm^{-1})	1000	800	750
λ_{IC}^{10} (cm^{-1})	200	108	550
λ_{IVR}^{2} (cm^{-1})	300	290	150
λ_{IVR}^{1} (cm^{-1})	100	110	75
λ_{IVR}^{0} (cm^{-1})	50	12	21
$d_1^{(01)}$	1.0	0.4	0.0
$d_2^{(01)}$	1.0	0.4	0.0
$d_1^{(12)}$	1.0	0.77	0.90
$d_2^{(12)}$	1.0	0.77	0.87
$d_1^{(02)}$	0.7	0.78	0.77
$d_2^{(02)}$	0.7	0.78	0.92
$d_1^{(1n)}$	n.a.	0.35	0.31
$d_2^{(1n)}$	n.a.	0.33	0.34
$\Delta\omega^{(02)}$ (cm^{-1})	400	700	541
$\Delta\omega^{(1n)}$ (cm^{-1})	n.a.	600	510
ampl. (02) / ampl. (1n)	n.a.	0.93	1.3

n.a.='not applicable';
"line-shape" parameters are shown in black; "dynamic" parameters are shown in blue

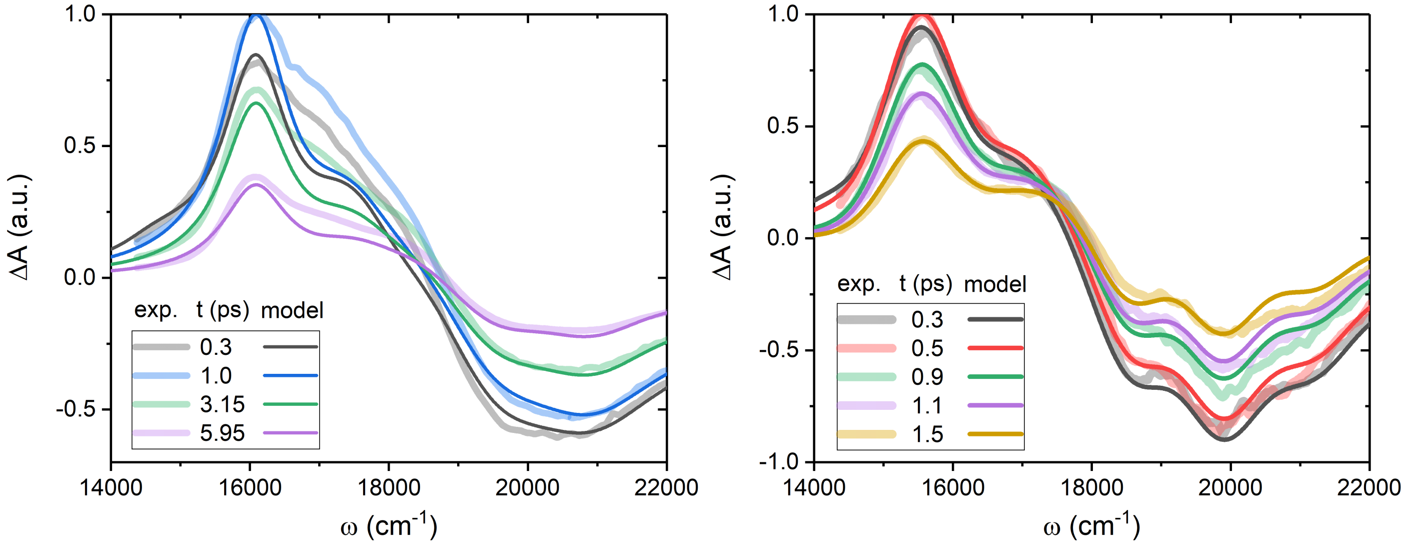

Figure S2 TA of Ctx and Rdx at early/intermediate times. Experimental results are given by thick faint lines, model fitting results are given by the full narrow lines.

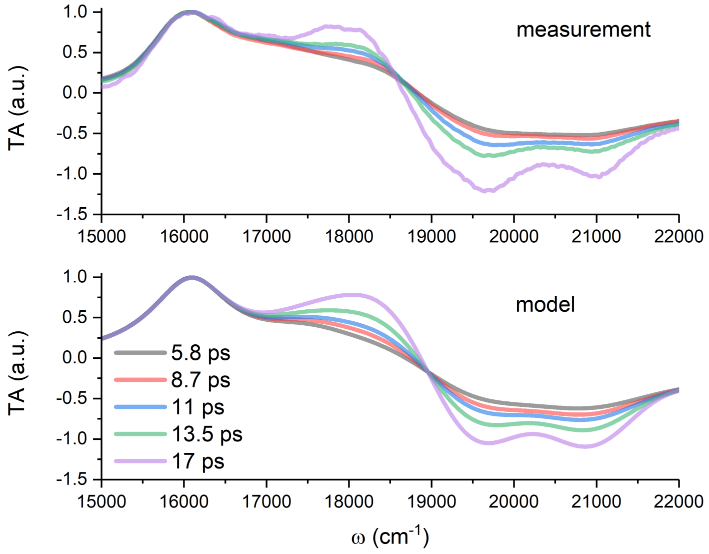

Figure S3 Narrowing of GSB signal in Ctx. Spectra are normalized to the maximum of IA from the $|S_1\rangle$ state.

are given in Table S2.

3 Molecular dynamics

Molecular dynamics (MD) simulations have been performed on Ctx, Rdx in benzene and, additionally, zeaxanthin (Zea) in THF in order to explore the structure of typical solvation shells of carotenoids.

3.1 Simulation details

Sample preparation. Ctx, Rdx and Zea were optimized in vacuum using the Gaussian 09 package[4] with a B3LYP/6-311(2d,p) basis set. MD were conducted using AMBER 16 package[5]. A 70 x 70 x 70 Å^3 cubic periodic solvent boxes were created around the carotenoids, where the solvent could be benzene (for Ctx/Rdx) or THF (for Zea), using xleap program embedded in AMBER. The total vdw box size was around 156 x 132 x 132 Å^3, depending on the particular solute and solvent combination. For all simulations General Amber Force Field (GAFF)[6] was applied for both carotenoids and solvents. The Coulomb cutoff was set to 8. No restraints were applied to the systems.

Equilibration/Relaxation. All simulations were minimized in energy with the steepest descend method for 500 steps followed by same steps of conjugate gradient method. The system was then relaxed and equilibrated in the constant density and temperature (NVT) ensemble and the constant pressure (NPT) ensemble with a time step of 2 femtosecond for 20 ps each, using Langevin thermostat with a target temperature of 300 K.

Production trajectories. The production runs were all performed with a 2 fs time step and a constant temperature of 300 K at an NVT ensemble to make analysis easier. To make sure the system is fully equilibrated, an additional 1 ns NPT run was conducted before the actual NVT production run.

3.2 Radial distribution function and solvation shells

In the three panels of Fig. S4 we show the RDFs for different carotenoid/solvent combinations: Ctx in benzene (left panel); Rdx in benzene (middle panel), zeaxanthin (Zea) in THF (right panel). Apparently, the results are similar for all segments for all carotenoid-solvent combination. Head groups tend to have larger radius in general, and RDF for the head group in Rdx is not satisfactorily resolved, yet the general trend is clearly observed.

To give a better impression of the shell structure, in Fig. S5 we show the distribution of the number of neighboring solvents within the given distance from the carotenoid (at this point, in order to avoid double counting, the whole molecule is considered rather than just segments). The counts are over different snapshots from the MD trajectory. The distributions of neighboring solvents within 5 and 6 angstroms are shown in green and orange, respectively.

3.3 Robustness of the model with respect to the FSS parameter variation (for Rdx)

FIg. S6 shows the modeled Rdx TA spectra (thin full/dashed/dotted lines) for various parameter values around the determined ones (shown by thin full lines); thick faint lines show the experimental results for reference. From left to right τ_{SC}, τ_{ss} and N_S are varied respectively.

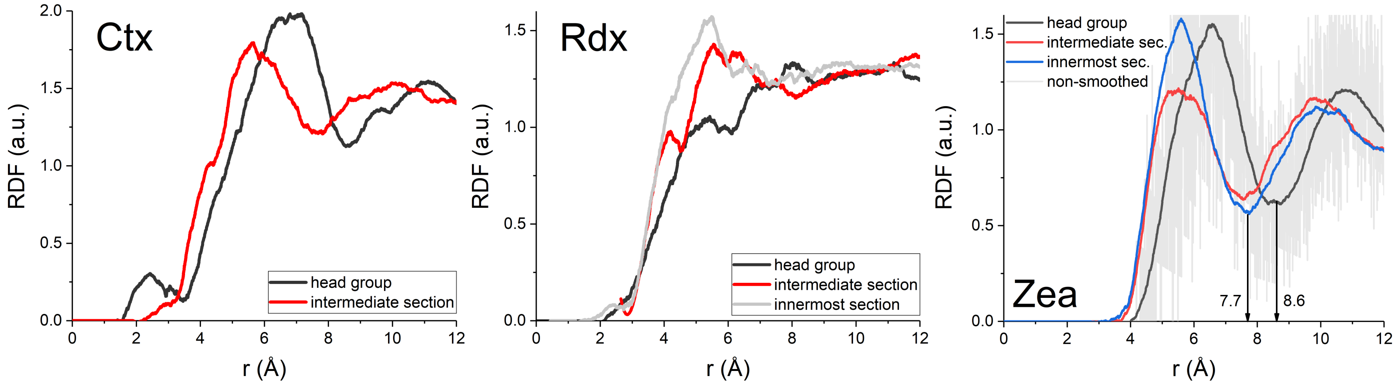

Figure S4 Radial distribution function of carotenoid segments. The segments are shown in the main text. Since the results show a two-fold symmetry (following the approximate molecular symmetry) only results for three segments are shown at most: the head group (outermost segment), intermediate section, innermost section. Results are shown after signal smoothing (Savitzky-Golay algorithm); on the right panel non-smoothed RDF is show in faint gray for reference (corresponds to the head group).

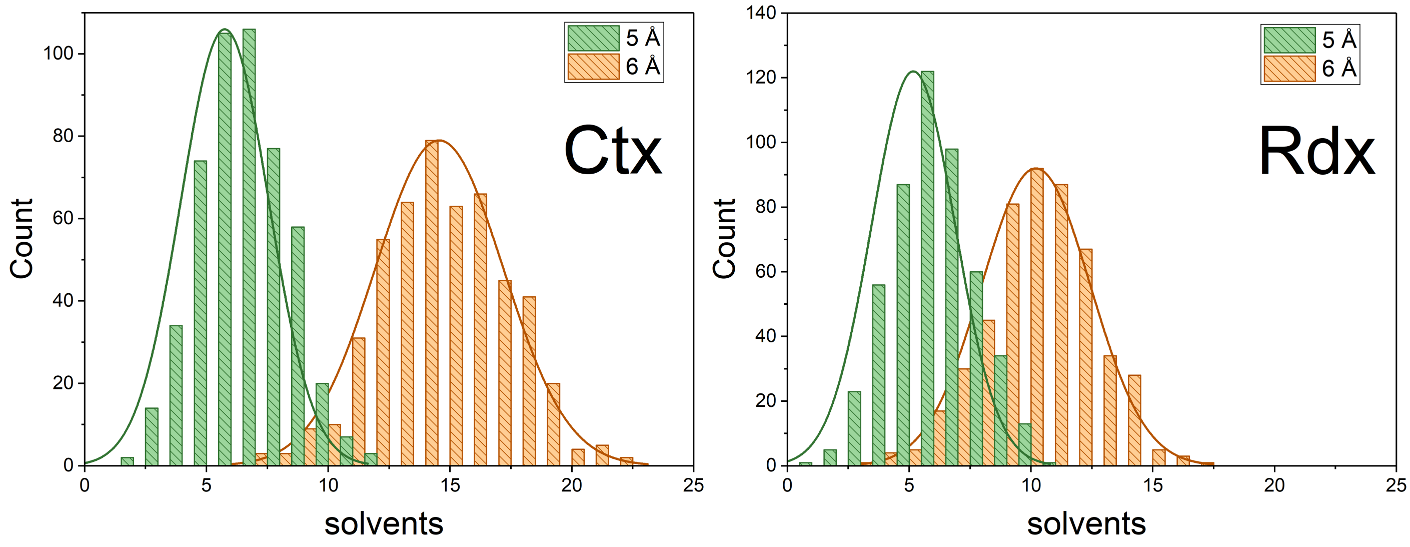

Figure S5 Distribution of nearest-neighbor solvents within the given distance from the full carotenoid (not segments). Left panel shows results for Ctx; right panel shows results for Rdx.

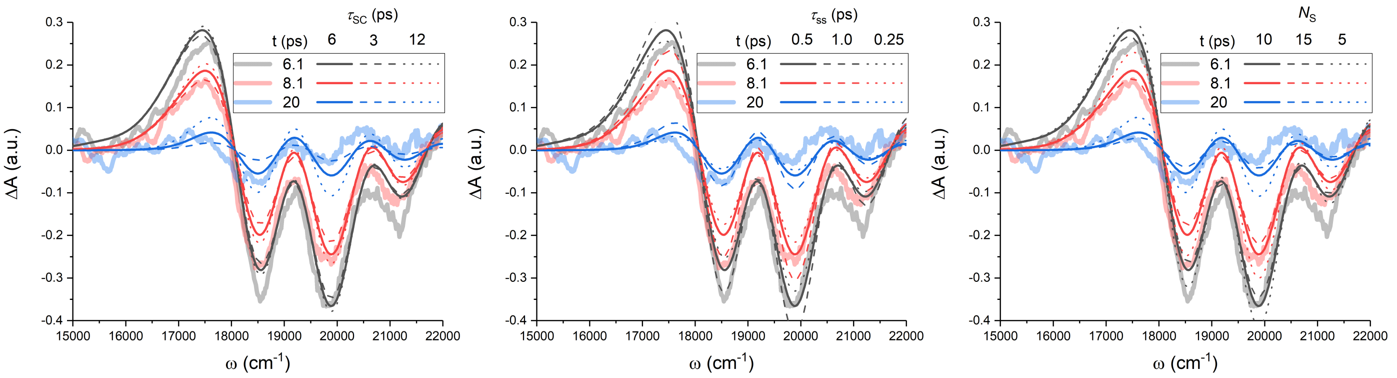

Figure S6 Effect of variation of FSS parameters to the model spectra. The TA spectra using the externally determined parameters are shown by thin full lines; thick faint lines show the experimental results.

Table S2 Normal mode frequencies of Ctx and Rdx.

No.	Ctx	Rdx	No.	Ctx	Rdx	No.	Ctx	Rdx	No.	Ctx	Rdx
1	4.9207	5.2759	70	507.2762	515.6758	139	1131.1647	1165.1996	208	1507.654	1557.706
2	9.535	8.4185	71	508.8608	522.5542	140	1153.565	1173.6131	209	1507.6562	1562.4568
3	11.3257	10.6225	72	522.9857	531.5254	141	1153.736	1187.7485	210	1514.5818	1581.744
4	12.9458	17.0519	73	535.4988	540.1254	142	1174.6936	1188.2337	211	1514.606	1600.845
5	21.7802	17.7506	74	538.0077	544.042	143	1180.1996	1194.1171	212	1554.9352	1606.7467
6	25.7552	22.7996	75	542.4216	549.5774	144	1188.115	1194.5861	213	1568.8066	1618.6466
7	34.2777	23.5367	76	567.4829	554.5732	145	1191.9222	1212.5247	214	1584.5158	1620.8473
8	39.8518	28.7601	77	567.6079	556.8913	146	1192.0361	1213.0727	215	1595.7518	1632.0731
9	44.8319	36.7746	78	567.9368	593.5759	147	1211.6972	1215.3979	216	1607.602	1639.3615
10	51.1157	48.4714	79	578.006	594.9078	148	1223.196	1215.5469	217	1616.507	1649.5908
11	55.4334	52.9093	80	602.4259	609.736	149	1223.4399	1230.7163	218	1624.5098	1663.0584
12	58.8374	54.8501	81	610.1687	613.8034	150	1224.2104	1242.5801	219	1642.7172	1735.6982
13	64.1923	80.1084	82	639.9422	644.0339	151	1229.7736	1249.2348	220	1647.5042	1736.3948
14	76.5689	81.0122	83	640.2622	644.4314	152	1233.2612	1264.3073	221	1658.1881	3007.2916
15	78.23	89.0537	84	651.9941	651.6437	153	1249.2974	1265.8125	222	1663.4778	3007.6952
16	79.4076	91.4675	85	672.2747	676.7582	154	1250.8596	1272.6448	223	1723.5579	3024.0197
17	91.5416	99.9689	86	716.6968	689.6153	155	1250.967	1274.1097	224	1723.8629	3024.9625
18	95.5393	101.3649	87	717.9214	690.2261	156	1287.3495	1292.6855	225	3012.3808	3026.9253
19	115.5448	102.7131	88	719.3249	767.5388	157	1287.4515	1293.4816	226	3012.3887	3026.9948
20	122.0506	111.0525	89	720.3216	767.6343	158	1299.9318	1310.0536	227	3022.5228	3027.9416
21	127.3626	113.2885	90	776.5762	830.2617	159	1300.4557	1317.2929	228	3022.5234	3028.1387
22	140.1746	115.576	91	776.6117	837.1923	160	1303.7558	1320.1504	229	3023.3585	3028.1607
23	142.0697	139.2254	92	831.2792	839.0129	161	1307.663	1334.4284	230	3023.4856	3028.3443
24	148.5394	141.0597	93	841.5975	850.4575	162	1324.2704	1335.1192	231	3024.7905	3037.9348
25	155.1327	152.6376	94	853.2542	852.4347	163	1333.9021	1336.514	232	3024.7918	3038.4205
26	165.9598	160.7451	95	856.6014	857.4394	164	1339.5099	1341.8608	233	3025.5464	3067.586
27	171.5461	162.6586	96	866.0999	863.596	165	1343.0036	1344.5133	234	3025.796	3067.6322
28	174.8207	169.0811	97	872.5336	865.1834	166	1354.0194	1362.0258	235	3025.9766	3069.0154
29	187.747	169.8009	98	877.8085	869.0333	167	1354.2969	1363.1598	236	3025.9802	3069.0237
30	197.9375	187.4775	99	882.0868	875.0423	168	1354.6358	1374.5151	237	3030.8091	3075.8661
31	203.884	203.5525	100	885.33	880.7703	169	1355.4762	1387.2135	238	3030.8194	3075.9258
32	213.5342	215.6366	101	894.7728	891.4559	170	1362.069	1387.5802	239	3049.2764	3083.0897
33	216.5634	218.6965	102	894.8786	907.7259	171	1362.0965	1398.835	240	3049.2941	3084.1956
34	217.2146	229.5934	103	902.765	907.828	172	1381.0548	1401.7923	241	3063.036	3086.6787
35	226.1226	231.2789	104	902.9393	915.5307	173	1381.1875	1404.345	242	3063.0361	3087.1676
36	228.4496	234.7926	105	909.0114	916.6397	174	1392.3888	1405.0531	243	3065.6228	3092.8821
37	233.9925	240.6246	106	920.3009	919.8612	175	1397.5848	1410.0486	244	3065.6666	3093.1225
38	234.8472	242.2985	107	920.8916	920.5765	176	1403.5013	1410.6751	245	3083.3681	3097.5409
39	241.4455	256.4633	108	922.4389	925.5882	177	1403.5101	1422.2012	246	3083.372	3098.2261
40	246.8352	259.0514	109	924.0112	926.3383	178	1403.5483	1423.0742	247	3084.4176	3114.2786
41	271.4804	262.4612	110	932.2356	929.6445	179	1410.9621	1424.4758	248	3084.4286	3115.0785
42	273.2533	264.4355	111	942.4771	931.5497	180	1412.7306	1425.3447	249	3087.2749	3118.0623
43	273.3246	266.0009	112	942.479	943.934	181	1412.7373	1428.722	250	3087.2899	3118.3987
44	293.7124	283.5043	113	982.9868	944.614	182	1425.4086	1432.1631	251	3087.5857	3123.6588
45	298.8513	285.4491	114	987.5324	951.5793	183	1425.4461	1434.8683	252	3087.6307	3124.0446
46	299.7517	308.1561	115	996.2666	961.8455	184	1427.314	1435.966	253	3100.5844	3124.5332
47	315.9859	309.3911	116	997.1874	962.2593	185	1427.4107	1462.317	254	3100.5855	3125.3251
48	316.1148	322.9296	117	1000.2619	990.4473	186	1430.7317	1463.4987	255	3104.8023	3126.6132
49	319.3642	326.1901	118	1004.25	992.367	187	1434.1197	1484.3351	256	3104.8157	3127.4436
50	319.6852	340.0694	119	1005.1388	1010.3607	188	1469.7046	1484.9848	257	3117.5321	3131.3888

No.	Ctx	Rdx	No.	Ctx	Rdx	No.	Ctx	Rdx	No.	Ctx	Rdx
51	341.3031	340.5827	120	1015.0308	1010.3962	189	1469.7061	1487.3203	258	3118.5401	3131.5805
52	354.0058	357.8829	121	1015.2998	1015.2659	190	1479.7698	1487.4822	259	3120.7166	3134.8287
53	358.0166	361.3576	122	1029.328	1023.0131	191	1479.9169	1489.2867	260	3121.0842	3135.2663
54	359.6946	364.2119	123	1029.9943	1024.0704	192	1486.1811	1489.5393	261	3126.0329	3141.9237
55	370.6613	365.9637	124	1034.9847	1040.3833	193	1486.2565	1489.7188	262	3126.8973	3152.7311
56	373.0349	369.3657	125	1035.0847	1041.4748	194	1486.3842	1490.0853	263	3128.2687	3170.1984
57	378.6063	377.7747	126	1044.7622	1045.1843	195	1486.483	1493.056	264	3128.2744	3170.4739
58	389.3916	391.6487	127	1044.7987	1046.1172	196	1487.7085	1493.4434	265	3130.3308	3177.569
59	389.8862	393.081	128	1050.8461	1056.5681	197	1487.8698	1495.7151	266	3130.3442	3177.7233
60	396.633	407.1046	129	1051.0588	1056.7851	198	1490.3547	1496.0034	267	3130.8003	3201.7579
61	399.4445	408.1005	130	1057.5405	1057.7418	199	1494.0297	1496.9828	268	3131.678	3202.3739
62	399.5675	452.1119	131	1058.0074	1058.2006	200	1494.0903	1497.1068	269	3145.7778	3214.7087
63	441.9204	462.0613	132	1059.1783	1058.791	201	1495.8179	1497.2996	270	3145.7834	3216.4211
64	447.6562	465.7481	133	1059.8339	1059.7392	202	1495.9067	1498.0494	271	3149.4052	
65	458.8119	484.6291	134	1060.2726	1063.2371	203	1499.109	1508.3929	272	3158.5134	
66	460.4629	492.6206	135	1060.3312	1063.8538	204	1499.393	1508.9819	273	3165.7184	
67	473.9693	494.7594	136	1113.9873	1129.2767	205	1500.0772	1522.8587	274	3165.7612	
68	488.9532	502.1718	137	1114.2263	1130.9911	206	1500.7988	1524.4484	275	3176.762	
69	492.2432	509.4386	138	1131.0392	1160.977	207	1500.8239	1554.285	276	3176.776	

References

1 L. Valkunas, D. Abramavicius and T. Mančal, *Molecular Excitation Dynamics and Relaxation*, Wiley-VCH, Weinheim, 2013.

2 T. Lenzer, F. Ehlers, M. Scholz, R. Oswald and K. Oum, *Phys. Chem. Chem. Phys.*, 2010, **12**, 8832–883.

3 V. Balevičius Jr., C. N. Lincoln, D. Viola, G. Cerullo, J. Hauer and D. Abramavicius, *Photosynthesis Research*, 2017, **135**, 55–64.

4 M. J. Frisch, G. W. Trucks, H. B. Schlegel, G. E. Scuseria, M. A. Robb, J. R. Cheeseman, G. Scalmani, V. Barone, B. Mennucci, G. A. Petersson, H. Nakatsuji, M. Caricato, X. Li, H. P. Hratchian, A. F. Izmaylov, J. Bloino, G. Zheng, J. L. Sonnenberg, M. Hada, M. Ehara, K. Toyota, R. Fukuda, J. Hasegawa, M. Ishida, T. Nakajima, Y. Honda, O. Kitao, H. Nakai, T. Vreven, J. A. Montgomery, Jr., J. E. Peralta, F. Ogliaro, M. Bearpark, J. J. Heyd, E. Brothers, K. N. Kudin, V. N. Staroverov, R. Kobayashi, J. Normand, K. Raghavachari, A. Rendell, J. C. Burant, S. S. Iyengar, J. Tomasi, M. Cossi, N. Rega, J. M. Millam, M. Klene, J. E. Knox, J. B. Cross, V. Bakken, C. Adamo, J. Jaramillo, R. Gomperts, R. E. Stratmann, O. Yazyev, A. J. Austin, R. Cammi, C. Pomelli, J. W. Ochterski, R. L. Martin, K. Morokuma, V. G. Zakrzewski, G. A. Voth, P. Salvador, J. J. Dannenberg, S. Dapprich, A. D. Daniels, . Farkas, J. B. Foresman, J. V. Ortiz, J. Cioslowski and D. J. Fox, *Gaussian-09 Revision D.01*, Gaussian Inc. Wallingford CT 2013.

5 D. Case, R. Betz, D. Cerutti, T. Cheatham, III, T. Darden, R. Duke, T. Giese, H. Gohlke, A. Goetz, N. Homeyer, S. Izadi, P. Janowski, J. Kaus, A. Kovalenko, T. Lee, S. LeGrand, P. Li, C. Lin, T. Luchko, R. Luo, B. Madej, D. Mermelstein, K. Merz, G. Monard, H. Nguyen, H. Nguyen, I. Omelyan, A. Onufriev, D. Roe, A. Roitberg, C. Sagui, C. Simmerling, W. Botello-Smith, J. Swails, R. Walker, J. Wang, R. Wolf, X. Wu, L. Xiao and P. Kollman, *AMBER16*, University of California, San Francisco 2016.

6 J. Wang, R. M. Wolf, J. W. Caldwell, P. A. Kollman and D. A. Case, *J. Comput. Chem.*, 2004, **25**, 1157–1174.
